# Multiple markers, niche modelling, and bioregions analyses to evaluate the genetic diversity of a plant species complex

**DOI:** 10.1186/s12862-017-1084-y

**Published:** 2017-11-29

**Authors:** Ana Lúcia A. Segatto, Maikel Reck-Kortmann, Caroline Turchetto, Loreta B. Freitas

**Affiliations:** Laboratory of Molecular Evolution, Department of Genetics, Universidade Federal do Rio Grande do Sul, P.O. Box 15053, Porto Alegre, RS 91501-970 Brazil

**Keywords:** Adaptive radiation, Hybridization, Pampas, Phylogeography, Solanaceae, Speciation

## Abstract

**Background:**

The classification of closely related plants is not straightforward. These morphologically similar taxa frequently maintain their inter-hybridization potential and share ancestral polymorphisms as a consequence of their recent divergence. Under the biological species concept, they may thus not be considered separate species. The *Petunia integrifolia* complex is especially interesting because, in addition to the features mentioned above, its taxa share a pollinator, and their geographical ranges show multiple overlaps. Here, we combined plastid genome sequences, nuclear microsatellites, AFLP markers, ecological niche modelling, and bioregions analysis to investigate the genetic variability between the different taxa of the *P. integrifolia* complex in a comprehensive sample covering the entire geographical range of the complex.

**Results:**

Results from molecular markers did not fully align with the current taxonomic classification. Niche modelling and bioregions analyses revealed that taxa were associated with different ecological constraints, indicating that the habitat plays an important role in preserving species boundaries. For three taxa, our analyses showed a mostly conserved, non-overlapping geographical distribution over time. However, for two taxa, niche modelling found an overlapping distribution over time; these taxa were also associated with the same bioregions.

**Conclusions:**

cpDNA markers were better able to discriminate between *Petunia* taxa than SSRs and AFLPs. Overall, our results suggest that the *P. integrifolia* complex represents a continuum of individuals from distant and historically isolated populations, which share some morphological traits, but are established in four different evolutionary lineages.

**Electronic supplementary material:**

The online version of this article (10.1186/s12862-017-1084-y) contains supplementary material, which is available to authorized users.

## Background

The delimitation of taxa at lower taxonomic levels is one of the greatest challenges of systematic biology [1], mainly due to the presence of highly conserved morphological traits [2] or morphological variation that does not follow traditionally defined species boundaries [3]. There are thus major open questions around the identity of species, the way to define them, and even the usefulness of the species concept in the context of conservation biology.

Closely related and young taxa frequently show morphological similarities and low levels of genetic differentiation due their evolutionary proximity. In this context, a species can be defined following different approaches: (1) tree-based methods define a species as a historical lineage according to phylogenetic concepts [4, 5]; (2) non-tree-based methods have been employed to uncover genetic structure associated with population isolation vs. species boundaries [6], which portrays aspects of population genetics that usefully elucidate the emergence of the species [7]; finally, (3) morphological distinctiveness can be used as a criterion to distinguish species from one another [8]. Importantly, to understand speciation in an evolutionary context, it is necessary to identify the factors that triggered the divergence process [9, 10]. In the case of the adaptive radiation of plants, the influence of different pollinators may be significant [11, 12]. Adaptive radiation drives the evolution of different forms in response to different environmental conditions or habitats, thus often resulting in high levels of morphological or taxonomical diversity, combined with low genetic differentiation between related taxa [13]. On the other hand, many morphs or ecotypes are associated with geographically distant habitats, where reproductive isolation is a result of the physical separation, rather than arising from biological constraints. In such cases, the partial or full reduction of the gene flow between newly established and ancestral populations could allow the independent evolution and differentiation of their gene pools over time, without necessarily leading to reproductive incompatibility [10]. As a consequence, the sustained potential for inter-hybridization prevents the use of the biological species concept to define these taxonomical entities.

The plant genus *Petunia* Juss (Solanaceae) is recognised worldwide thanks to its prominent member *Petunia hybrida* (Hook.) Vilm, a widely cultivated interspecific hybrid considered to be a “supermodel” plant [14]. *Petunia hybrida* resulted from crosses between the white-flowered *P. axillaris* (Lam.) Britton, Sterns & Poggenb and *P. interior* T. Ando & Hashim as its main purple ancestor [15]. *Petunia* comprises 14 diploid species [16], predominantly native to southern Brazil, with the exception of two taxa (*P. occidentalis* R. E. Fr. and *P. axillaris* ssp. *subandina* T. Ando) that are found in the pre-Andean mountains in northern Argentina [17]. Two centres of diversity have been described for this genus [16]. Both of these represent transitional areas between tropical and subtropical formations and form a boundary for the distribution of many plant species: they represent a northern limit for many Austral-Antarctic elements (corresponding to the lowland grasslands of the Pampas region in Uruguay, the Argentinean province of La Pampa, and the Brazilian state of Rio Grande do Sul) and a southern limit for predominantly tropical elements (corresponding to the open highland fields of the Brazilian states of Rio Grande do Sul and Santa Catarina). Transitional areas concentrate a significant fraction of adaptive variation and are therefore an ideal setting for the study of speciation [18].

Phylogenetic analyses based exclusively or predominantly on plastid DNA (cpDNA) markers have revealed two major clades of *Petunia* species associated with elevation, a highland (> 500 m) and a lowland (< 500 m) clade [19–21]. On the other hand, studies using mostly nuclear markers have found two clades associated with corolla morphology (short vs. long corolla tube; Additional file 1: Figure S1F) [22–25]. Phylogeographic studies of *Petunia* species sharing morphological, ecological or geographical traits have confirmed an extremely recent divergence of *Petunia* species and are indicative of a diversification following colonization processes related to climate changes that took place during the Pleistocene [21, 26–31]. Moreover, the occurrence of artificial [32] and natural [26, 29, 33] hybrids between *Petunia* species suggest that intrinsic barriers to interspecific hybridization do either not exist, or else are rather weak. Where these barriers do exist, they are mainly prezygotic, and interspecific *Petunia* crosses yield viable diploid offspring, at least under controlled conditions [14]. Despite the apparent morphological and ecological diversity found within the genus *Petunia*, molecular studies have shown very low levels, or even an absence, of genetic diversity between species, a hallmark of recent adaptive radiation [19, 20, 22, 24].

The taxa of the *Petunia integrifolia* (Hook.) Schinz & Thell complex are delimited by few specific morphological traits (Additional file 1: Figure S1 A-E; Additional file 2: Box S1; Additional file 3: Box 2). All its taxa are bee-pollinated, and the ranges of several of them are geographically close, with some overlaps [34]. Specimens with an intermediary morphology have been observed between taxa of this complex; however, they have not been characterised as hybrids using morphological or molecular traits [35]. It thus remains unknown whether these intermediary morphological traits represent intra-population variation or result from hybridization. In general, the *Petunia integrifolia* complex is characterised by a purple and infundibuliform corolla, violaceous pollen, and a stigma that is located between the anthers of the didynamous stamens. Due to the many morphological similarities across the *P. integrifolia* complex, there is little agreement on the number and taxonomic status of its taxa [16, 34, 36] (Additional file 2: Box S1).

**Fig. 1:**
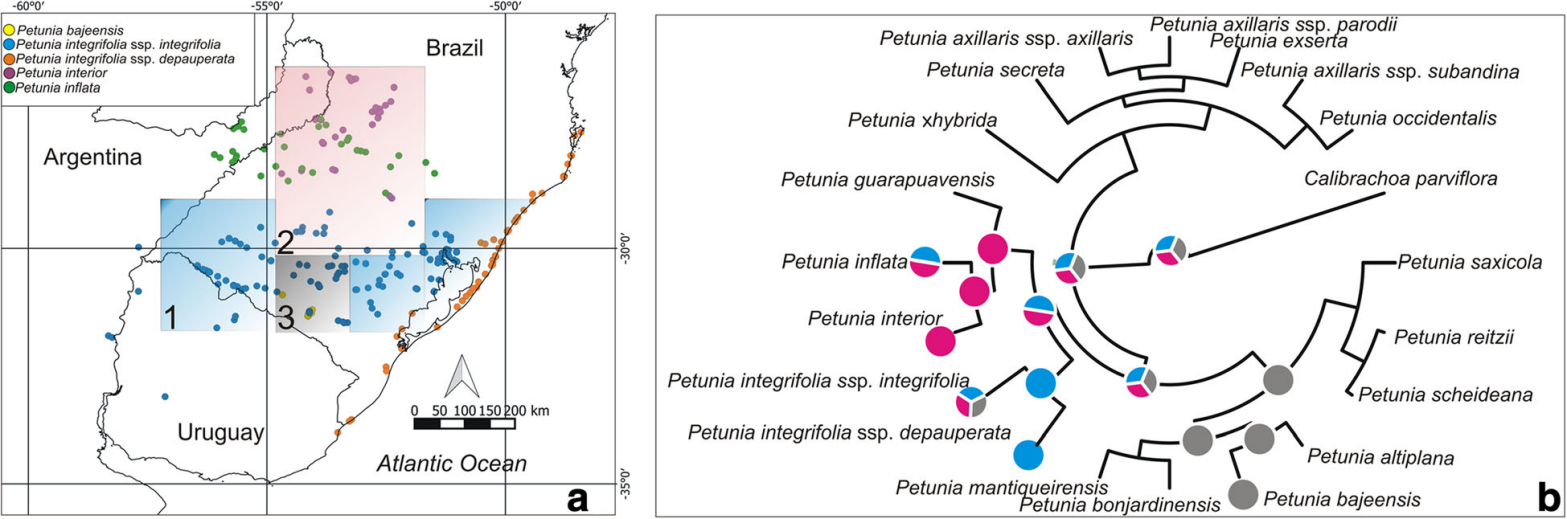
Geographical distribution and bioregion analysis of the species of the *P. integrifolia* complex. **a** Taxon distribution based on field collection and data available at SpeciesLink (http://www.splink.org.br) and the Global Biodiversity Information Facility (GBIF; http://www.gbif.org). Bioregions delimited according to Infomap Bioregions [79] are shown in transparent boxes (blue: bioregion 1; pink: bioregion 2; grey: bioregion 3. **b** Phylogenetic tree of *Petunia* species based on sequences from Segatto et al. (2016) with ancestral ranges reconstructed under Fitch’s method of Maximum Parsimony. Pie charts depict the most parsimonious ancestral range at selected nodes, and colours are according to the map bioregions (Fig. 2a). Country boundaries obtained from http://www.diva-gis.org

Here, we define the *P. integrifolia* complex to include all taxa that share morphological traits with *P. integrifolia*, as well as those that have been classified as subspecies of *P. integrifolia* at least once: *P. bajeensis* T. Ando & Hashim, *P. inflata* R. E. Fr., *P. integrifolia* Ss*p. depauperata* R. E. Fr. (hereafter, *P. depauperata*), *P. integrifolia* Ss*p. integrifolia* (hereafter *P. integrifolia*), and *P. interior* (for a morphological overview see Additional file 1: Figure S1A-E and Additional file 3: Box S2). A number of authors also consider *P. littoralis* L. B. Sm. & Downs and *P. riograndensis* T. Ando & Hashim to be valid species in this group. However, it has been suggested to synonymise *P. littoralis* as *P. depauperata*, as a result of their morphological similarities and geographical distribution [36]. For the same reasons, *P. riograndensis* has also been synonymised as *P. integrifolia* [16]. The grouping of these pairs of taxa (*P. littoralis* + *P. depauperata* and *P. riograndensis* + *P. integrifolia*) is fully supported by the phylogeographic analysis of molecular data [27, 28], thus justifying the synonyms; however, using a phylogenetic approach, a different scenario has been proposed [25]. In this work, we consider these taxa as synonymous, as suggested previously [16]. Indeed, our analyses did not find any differences to justify their separation. Phylogenetic analyses of the *P. integrifolia* complex have repeatedly returned incongruent results, and a comprehensive phylogeographic study evaluating the level of differentiation between the remaining species, *P. bajeensis*, *P. inflata*, and *P. interior*, is lacking. The patterns of geographic distribution are highly variable across the taxa of the *P. integrifolia* complex. They include the restricted and narrow distribution of *P. bajeensis*, a pattern of wide distribution across specific biogeographic provinces, as seen in *P. depauperata*, and the general wide distribution of *P. integrifolia* (Fig. 1a). Generally, the taxa of the *P. integrifolia* complex are distributed across areas characterised by ecological differences. Paired with geographical and historical barriers, these ecological differences have promoted the establishment of distribution boundaries: *P. bajeensis* and *P. integrifolia* are found in the Pampas, *P. depauperata* is endemic to the South Atlantic Plain Coast, where it grows in salty and nutritionally poor soils (also in the Pampas), and *P. inflata* and *P. interior* are native to the Paraná province [34, 37, 38].

Here, we evaluated the genetic variability of the *P. integrifolia* complex, based on a comprehensive sample of taxa and individuals per taxon, which covers most of the geographical range and the complete contact zone for all taxa. We used a molecular approach based on non-coding cpDNA markers and polymorphic nuclear markers (SSR and AFLP), in tandem with ecological niche modelling and bioregions analyses. We sought to answer the following questions: (1) Does the current taxonomy of the *P. integrifolia* complex match the molecular diversity?; (2) Are the different markers equally effective in assigning individuals to morphological taxa?; and (3) Can ecological niche modelling explain the current species distribution as a result of ancient or current isolation or contact?

## Methods

### Plant material

We studied five taxa belonging to the *P. integrifolia* complex. Taxa were identified according to Stehmann et al. [16] (for morphological characterization and taxon distinction see Additional file 3: Box S2). Our sample was collected mainly in southern Brazil. It covers most of the taxa’s distribution, while focusing on the main area of convergence of the *P. integrifolia* complex, where all five taxa are found. The geographical coordinates of all collection sites were obtained using a global positioning system (GPS) unit. We deposited vouchers at the BHCB (Universidade Federal de Minas Gerais, Belo Horizonte, Minas Gerais, Brazil) or ICN (Universidade Federal do Rio Grande do Sul, Porto Alegre, RS, Brazil) herbaria (see Additional file 4: Table S1).

This work was conducted under Brazilian Federal Government permit MP 2.186/16 for the access of plant genetic information to develop evolutionary or taxonomic studies. No specific collection permits were required because none of the analysed taxa are federally listed as endangered or protected, and because no collection sites were located within protected areas.

### cpDNA sequencing and analysis

Genomic DNA was extracted from dried young leaves following the CTAB protocol [39]. We analysed 26 individuals from three populations of *P. bajeensis*; 308 individuals from 69 populations of *P. depauperata*; 39 individuals from ten populations of *P. inflata*; 84 individuals from 15 populations of *P. interior*; and 114 individuals from 38 populations of *P. integrifolia*, making for a total of 571 individuals from 135 collection sites. The intergenic regions of cpDNA *trnG-trnS* and *psbA-trnH* were amplified, as previously described for *Petunia* species [26], using universal primers as described for each spacer [40, 41]. PCR products were purified using PEG 20% (polyethylene glycol; Sigma-Aldrich Co., St. Louis, MO, USA) [42] and sequenced in a MegaBACE1000 (GE Healthcare Bio Sciences Corp., Piscataway, NY, USA) automatic sequencer according to the manufacturer’s instructions and the DYEnamicET Terminator Sequencing Premix Kit (GE Healthcare). Some published sequences were also included in this work [15, 28]. All sequences were deposited in GenBank (see Additional file 4: Table S1). For each marker, both forward and reverse strands were checked using the Chromas 2.0 software (Technelysium, Helensvale, Australia), aligned with MEGA 6 [43] using the Clustal W algorithm, and manually edited when necessary. Poly-A/T regions and small inversions are generally highly variable and homoplasic [44, 45] and were thus not considered in our analyses. Contiguous insertions/deletions (indels) of more than one base pair (bp) were treated as single mutational events [46].

The haploid plastid genome does not normally undergo recombination. This uniparental transmission means that it is inherited as a unit, and the two cpDNA intergenic spacers were therefore concatenated and treated as a single sequence in all analyses. The numbers of variable and informative sites in the manually edited alignment were obtained from the output of MEGA 6. Haplotypes were identified using DNAsp 5.10.01 [47], and the relationships between haplotypes were estimated using the Median-Joining method [48] implemented in the Network 4.612 software [49]. Indices of haplotypic (h) and nucleotide (π) diversities [50] and inter- and intra-taxon genetic variation by means of analysis of molecular variance (AMOVA) were obtained using Arlequin 3.5.1.2 [51].

The dated haplotype phylogenetic trees were estimated by Bayesian inference as implemented in BEAST 1.8.4 [52]. Two independent runs were employed consisting of 1 × 10^8^ Markov chain Monte Carlo (MCMC) iterations, sampling every 1000 generations under the HKY (Hasegawa, Kishino, and Yano) nucleotide substitution model with four gamma categories as obtained in JModelTest according the Akaike information criterion (AIC) [53]. We used a lognormal relaxed clock, Yule process as tree prior, and the prior for nucleotide substitution rates was as following: a gamma distribution prior with a shape parameter 1.6 and scale parameter 1.6 × 10^−9^ as a prior, assuming an offset value of 1 × 10^−9^ s/s/y. This rate was obtained by compiling rates of plastid markers for shrubs and herbaceous plants with a generation time of up to three years [27]. Tracer 1.6 [54] was used to check for convergence of MCMC and adequately effective sample sizes (ESS > 200) after discarding the first 10% of generations as burn-in. The final joint sample was used to estimate the maximum clade credibility tree using the TreeAnnotator software, part of the BEAST package, setting 0.5 as limit of posterior probability. Statistical support for the clades was established by assessing the Bayesian posterior probability (PP) with node heights summarized to reflect the posterior median. The FigTree 1.4.0 software package [55] was used to draw and edit the phylogenetic tree. As an outgroup, we used haplotypes of *Calibrachoa excellens* (R.E.Fr.) Wijsman (Additional file 4: Table S1).

The demographic patterns of each taxon were assessed separately through a Bayesian Skyline Plot (BSP) [56], which considers historical changes in the effective size, as implemented in BEAST software. The priors for this analysis were the same as those used in the haplotype phylogenetic analysis, as previously described and including the sequences of all individuals in the analysis. The BSP was reconstructed using Tracer 1.6.

### Microsatellite genotyping and analysis

To estimate the genetic variability based on simple sequence repeats (SSR), we randomly selected individuals of all taxa of the *P. integrifolia* complex among those included into the cpDNA analysis. We sampled one population of *P. bajeensis* (B1), three populations of *P. depauperata* (D1, D25, and D30), *P. inflata* (I1, I3, and I6), and *P. interior* (T3, T6, and T8), and five populations of *P. integrifolia* (G6, G7, G8, G24, and G31), for a total of 108 individuals (Additional file 4: Table S1). These populations were selected to cover the entire geographical distribution of these taxa [15, 16, 28].

We amplified seven microsatellite loci originally isolated in *P. depauperata* [57], according to Segatto et al. [15]; the individuals used in that work formed part of the sample described above. We estimated allele number (A), allele richness (AR), gene diversity (GD), inbreeding coefficient (*F*_*IS*_), and exclusive alleles (EX) using Fstat [58]. We also evaluated the observed heterozygosity (H_O_), expected heterozygosity (H_E_), polymorphic information content (PIC), the proportion of null alleles (NUL), and the deviation from the Hardy-Weinberg equilibrium using Cervus 3.0.3 [59].

Intra- and interspecific genetic differentiation was assessed by AMOVA using Arlequin. Population subdivision was estimated through *F*-statistic analogues (*R*_*ST*_), and the statistical significance was determined based on 2 × 10^4^ permutations.

Bayesian clustering analysis, as implemented in Structure 2.3.3, was used to establish population structure and individual ancestry [60]. As gene flow was expected among the natural populations, an admixture model using correlated allele frequencies was included [61]. We approximated the optimum number of groups (K) by varying K from 1 to 10, to exceed the number of different possible taxa (five), and ran the analysis ten times for each K. Each run was performed using 2.5 × 10^5^ burn-in periods and 10^6^ MCMC repetitions after burn-in. The optimal number of genetic clusters was determined using the ΔK method [62], which favours the model with the greatest second-order rate of change in lnPr (X|K), as implemented in the online Structure Harvester [63]. The results for the best K value were summarized using Clumpp 1.1.2 based on the average pairwise similarity of individual assignments across runs with the Full-Search method, weighted by the number of individuals in each population, and G’ statistics [64]. Distruct 1.1 [65] was used to visualize the Structure results after processing with Clumpp.

### AFLP profiles and analysis

For the amplified fragment length polymorphism (AFLP) analyses, we randomly selected seven to 16 individuals per taxon among the individuals previously analysed for cpDNA, for a total of 51 individuals covering the entire geographic distribution of the *P. integrifolia* complex. AFLP analyses were carried out with *Eco*RI and *Mse*I (*Tru*I) enzymes (New England Biolabs, Hitchin, UK) and following standard protocols [66]. Initially, nine selective primer combinations of 5′-fluorophore-labelled *Eco*RI primers and unlabelled *Mse*I primers were tested. The best three pairs (Fam-*Eco*RI-AAC/*Mse*I-CAC, Hex-*Eco*RI-AAG/*Mse*I-CAC, and Ned-*Eco*RI-AGC/*Mse*I-CAG) were retained because they generated clear bands and high variability in preliminary tests for these taxa. The amplification products were subjected to capillary electrophoresis in an ABI 3500 XL automatic sequencer (Applied Biosystems, Foster City, USA). Fragments for each primer combination were processed and analysed through the GeneMapper 4.1 AFLP ® software (Applied Biosystems), which automatically generates presence (1) or absence (0) matrices for all bands. These matrices were then compared with the corresponding electropherograms. Bands in the 100–450 bp range were compared for all samples. The reliability of the data was evaluated by the comparison of duplicates, and about 10% of individuals fulfilled this purpose. The percentage of polymorphic loci (#P_loc) and Nei’s gene diversity (Hj) [67] were calculated within each species using AFLPsurv 1.0 [68]. To understand how genetic variation is partitioned within and among taxa, an AMOVA based on pairwise F_*ST*_ among taxa was carried out using Arlequin. Patterns of genetic clustering among individuals were verified in Structure applying the admixture model and the correlated allele frequencies, using the AFLP data as diploid and dominant data [61]. Each analysis was performed for 100,000 generations, with the first 10% discarded as burn-in. Analyses were conducted for K = 1 to 7, with five repeats for each K. The number of tested K values was reduced based on the previous results using SSRs and due to the long computational time of this analysis. Optimum numbers of genetic clusters, summary results, and visualization were as described above for SSRs.

Bayesian inference (BI) analysis was carried out in MrBayes 3.2 [69], to obtain a majority consensus tree, with F81-like as the evolutionary model for restriction sites [70] and equal rates as obtained in JModelTest, according AIC criterion, and using the coding bias option ‘*lset = noabsencesites*’. We ran a ten million generation chain, sampled at the length of every 1000 trees. The convergence was verified using TRACER 1.6 [54] after removing 10% of the sampling as burn-in.

### Niche modelling and biogeographic region analysis

Georeferencing for the collection sites of all taxa in the *P. integrifolia* complex was acquired through direct field observation, SpeciesLink [71], and the Global Biodiversity Information Facility [72]. Records obtained from the databases were verified manually for incongruences, and only those matching species distribution were kept (Additional file 5: Table S2). Only records with global positioning system coordinates and detailed localisation were used. This did not compromise the analysis because it still allowed a complete coverage of the taxa’s distribution. Explanatory variables included a set of 19 bioclimatic Raster layers at a 30 arc-second resolution (ca. 1 km^2^ at the equator) from the WorldClim website version 1.4 [73]. We carried out ecological niche modelling (ENM) for the current species distribution under three contrasting past climate conditions [Last Interglacial (LIG) - ~120,000–140,000 years before present (yBP); Last Glacial Maximum (LGM) – 21,000 yBP, and Mid-Holocene (MH) – 6000 yBP] using a model of maximum entropy (Maxent 3.3.3) [74]. The grid layers were cut so as to include the entire geographical distribution for all taxa and extracted through the RASTER package [75] implemented in R software [76]. Pearson’s correlation between the variables was calculated using the Raster package, and multi-collinearity was minimized by selecting 10 bioclimatic variables pairs showing an *R* > 0.75 and presenting the lowest percentage of importance to the model in a preliminary run [77, 78]. We used this approach in order to identify areas with high suitability that could have harboured the taxa through the Pleistocene climate changes and ecological variables that could explain the geographical differentiation among taxa.

To understand the role of the biogeographical regions in the evolution of the species, we used the web application Infomap Bioregions [79] to identify taxon-specific bioregions from our species distribution data, for discussion about terminology and concepts see Vilhena and Antonelli [80]. A species tree was used as input for the online INFOMAP BIOREGIONS software [81] to reconstruct the ancestral range for the *P. integrifolia* complex through Fitch’s method of maximum parsimony [82]. The *WUSCHEL* gene sequences from Segatto et al. [25] were used to construct the phylogenetic tree of the *Petunia* species, according to the classification of Stehmann et al. [16]. We used these markers because the phylogenetic tree obtained based on *WUSCHEL* sequences was the best supported among *Petunia* phylogenies and it is in agreement with previous works using nuclear and plastid markers [24]. We used BEAST 1.8.4 [52] applying the evolutionary model GTR with four gamma categories (obtained from JModelTest according AIC criterion), Yule speciation model, and a lognormal relaxed molecular clock to obtain the tree. Two runs of 10^8^ generations were carried out, sampling every 1000 generations, and the first 10% were discarded as burn-in. Markov chain convergence was ensured by values of ESS > 200, and the resulting plots were checked in Tracer. TreeAnnotator was used to select the maximum clade credibility tree. Statistical support for the branches was measured in Bayesian posterior probabilities (PP).

## Results

### cpDNA diversity

**Fig. 2:**
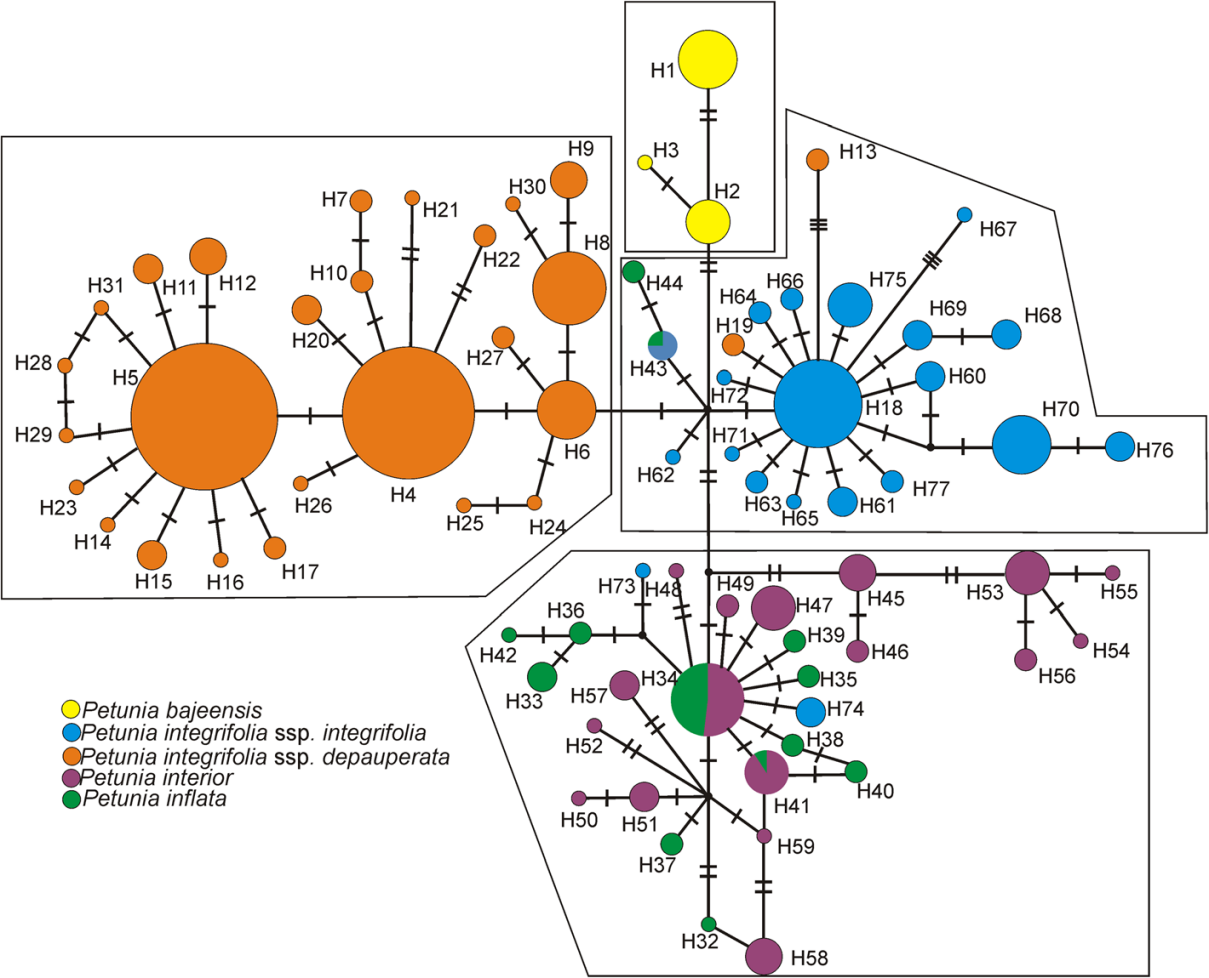
Median-joining network of concatenated plastid haplotypes coloured by taxon. Transverse lines indicate mutations. The four haplogroups referred to in the text are presented in boxes

The combined cpDNA sequence data (*trnG-trnS* and *psbA-trnH*) for the 571 individuals yielded an 1127 bp alignment, with 65 variable and 50 parsimoniously informative sites resulting in 77 haplotypes. The median-joining network grouped species into four main groups (Fig. 2). We found low haplotype sharing among species, with only three haplotypes shared between two species each (Additional file 6: Table S3; Fig. 2): H34 and H41 were shared by *P. interior* and *P. inflata*, whereas H43 was observed in *P. inflata* and *P. integrifolia*. The largest number of haplotypes (27) was found in *P. depauperata*, the most frequent ones being H4 (found in 110 individuals) and H5 (found in 85 individuals). The smallest number of haplotypes (three) was observed in *P. bajeensis*, the species with the smallest number of both collection sites and individuals, and with the most restricted geographical range among the analysed taxa. For both *P. interior* and *P. depauperata*, haplotype differentiation clearly revealed subgroups, with different genetic components in these species.

Overall, the haplotypic diversity was h = 0.93, and the nucleotide diversity was π = 0.32%. The highest values for haplotype and nucleotide diversity were observed for *P. interior* (h = 0.91 and π = 0.34%), the lowest for *P. bajeensis* (h = 0.52 and π = 0.09%). *Petunia depauperata* (h = 0.77 and π = 0.13%)*, P. integrifolia* (h = 0.84 and π = 0.18%)*,* and *P. inflata* (h = 0.86 and π = 0.24%) presented the intermediate values of diversity. AMOVA results (Table 1) indicated greater interspecific than intraspecific variation (~64% vs ~36%).

**Table 1:** AMOVA based on cpDNA haplotypes, microsatellites and AFLP profiles of the *Petunia integrifolia* complex

	Source of variation	Sum of squares	Variance component	Percentage of variation
cpDNA	Among groups	682.224	1.837	64.4
	Within groups	574.663	1.015	35.6
SSR	Among groups	534.059	2.660	38.6
	Within groups	779.188	4.234	61.4
AFLP	Among groups	1444.143	24.713	17.2
	Within groups	5348.117	118.847	82.8

**Fig. 3:**
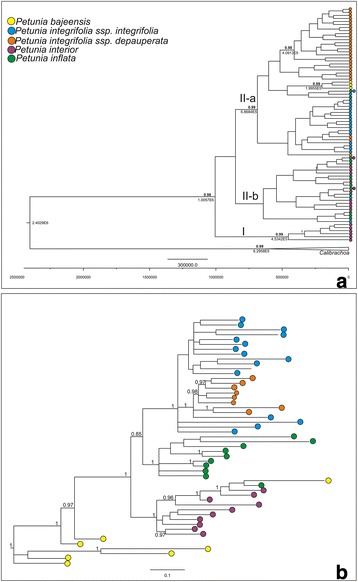
Phylogenetic trees based on (**a**) haplotypes and (**b**) AFLP markers. The two main groups shown on the Bayesian haplotype phylogenetic tree correspond to different grasslands locations (Group II-a, Pampas Grasslands; Group I and II-b Highland grasslands inside Atlantic rainforest). Posterior probabilities >0.90 are shown next to the clades. The AFLP phylogenetic tree (**b**) showed a different pattern

The phylogenetic tree based on cpDNA haplotypes revealed two primary groups: one well supported (PP = 0.99) corresponding to the haplotypes of *P. interior* 45, 46, 53, 54, 55, and 56 (I) and another not supported clade corresponding to the rest of the haplotypes (II). Clade II has two subclades, one well supported corresponding mainly to haplotypes of *P. depauperata* + *P. integrifolia* and *P. bajeensis* (IIa), and another group mainly including haplotypes of *P. inflata* and *P. interior* (IIb), and a divergence time ca. 687 thousand years ago (Kya; Fig. 3a). These two groups of species inhabit different environments, the first one is from the Pampas grasslands, the second from the grasslands of the Highland inside the Atlantic rainforest, which forms a natural mosaic with the *Araucaria* forest. Within each group, the analyses supported several subclades. In group IIa, *P. depauperata* formed one clade (PP = 0.98; ~409 Kya) which further divided the haplotypes into Central, North, and South groups. *P. bajeensis* also formed a cluster with evidence for recent diversification (PP = 0.99; ~199 Kya) (Fig. 3a).

The Bayesian Skyline Plot (Additional file 7: Figure S2) showed different patterns for each group of species forming the two main clades of the Pampas and Highland grasslands, respectively, indicating periods of expansion and contraction. For *P. bajeensis,* the plot suggested a smooth yet constant population decline until ~25 Kya, from when population growth was observed until the present. On the other hand, *P. integrifolia* and *P. inflata* showed a recent population decline starting around 5 Kya until present. *Petunia interior* experienced a slight decrease over time followed by a recent expansion. However, these results should be taken with caution because of the large credibility intervals associated with population size estimates.

### Microsatellite diversity and structure

We analysed seven SSR loci. Six of these were polymorphic in *P. bajeensis*, *P. integrifolia*, and *P. inflata;* five were polymorphic in *P. interior.* All seven loci were polymorphic in *P. depauperata* (Additional file 8: Table S4). The average number of alleles found per locus per taxon was two for *P. bajeensis*, three for *P. integrifolia* and *P. depauperata*, four for *P. inflata,* and five for *P. interior*. The locus PID1G6 was monomorphic in *P. bajeensis*, as was PID3G5 in *P. inflata* and *P. interior,* and PID4C6 in *P. integrifolia* and *P. interior*. Genetic diversity evaluated across all loci was generally higher in populations of *P. inflata* and *P. interior* than in the other species when estimated via allelic richness and gene diversity (Additional file 8: Table S4). In *P. bajeensis*, the inbreeding coefficient was negative when considering all loci, indicating an excess of heterozygotes. The other species showed negative values for one (*P. depauperata*) or two (*P. integrifolia*) loci and few positive values at the other loci. Private alleles were discovered at one locus for *P. integrifolia* and *P. inflata,* at two loci for *P. depauperata*, and three for *P. interior* (Additional file 8: Table S4). Only the locus PID1F1 in *P. interior* deviated from the Hardy–Weinberg equilibrium after a Bonferroni correction (*P* < 0.05; Additional file 9: Table S5). The frequency of null alleles was negative or close to zero for all loci calculated, suggesting an absence of null alleles (Additional file 9: Table S5). The AMOVA indicated that 38% of the genetic variation was a result of differences between taxa, while 62% was due to within-taxon variation (Table 1).

**Fig. 4:**
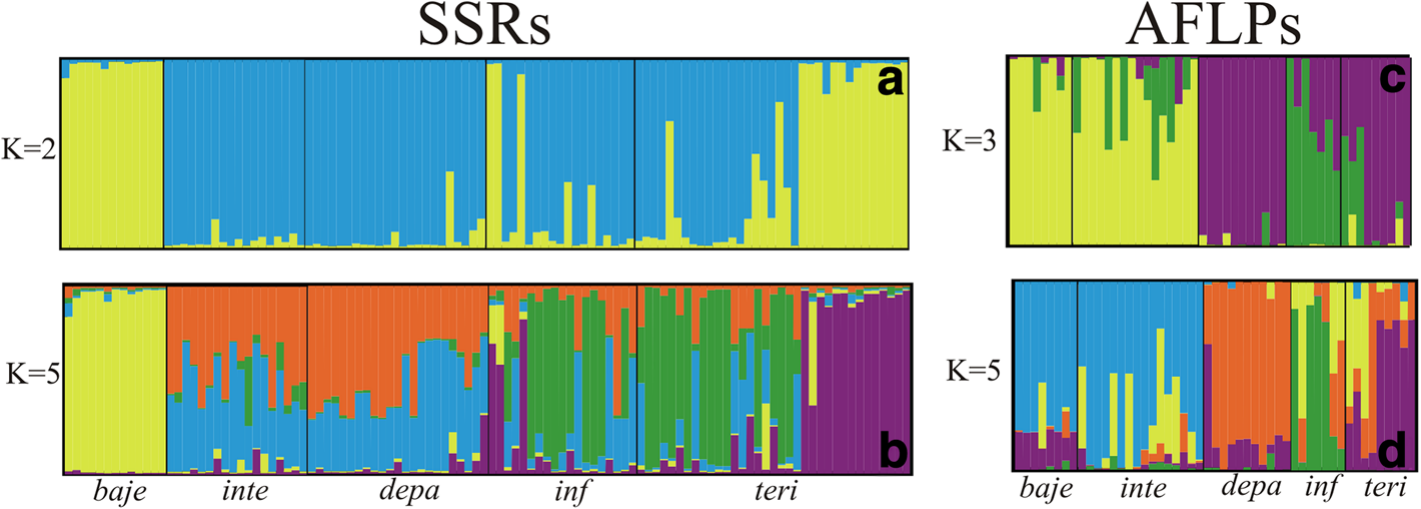
Results of the Structure analyses based on microsatellites and AFLP markers. Each individual is represented as a vertical line partitioned into K-coloured components that represent an individual’s proportional assignment to one of the genetic clusters for SSRs and K = 2 (**a**); for SSRs and K = 5 (**b**); for AFLPs and K = 3 (**c**); and for AFLPs and K = 5 (**d**). *Petunia bajeensis* (*baje*); *P. integrifolia* Ss*p. integrifolia* (inte); *P. integrifolia* Ss*p. depauperata* (*depa*); *P. inflata (inf*); *P. interior* (*teri*)

Based on the SSR data, we found the best value for K to be 2 in the Structure analysis (Fig. 4a), with the first cluster comprising *P. bajeensis* and various individuals of *P. interior* from a single collection site (T8), and the second one including all other species and the remaining populations of *P. interior*. For K = 5 (the number of morphological taxa included in our analyses; Fig. 4b), *P. bajeensis* showed a private genetic component. *Petunia interior* population T8 shared a genetic component with *P. inflata*; individuals of both *P. integrifolia* and *P. depauperata* had two different components, both of which were equally shared by both species. Finally, *P. inflata* and the remaining populations of *P. interior* showed three genetic components that did not distinguish them (one was exclusive to these taxa; the second was common to *P. integrifolia* and *P. depauperata*; the last was shared with the T8 population of *P. interior*).

### AFLP profiles and genetic structure

Both gene diversity values (Hj) and percentage of polymorphic loci (#P_loc) found for the five taxa of the *P. integrifolia* complex fell into a narrow range (Hj = 0.00427–0.00512; #P_loc = 51.4–60%; Additional file 10: Table S6). AMOVA results (Table 1) indicated higher levels of intraspecific than interspecific variation (~83% vs ~17%).

The best number of groups in the AFLP-based clustering analysis using Structure was K = 3 (Fig. 4c), with all three genetic components present in all taxa, albeit in different proportions. Individuals belonging to *P. interior* were divided into two groups, of which one was very similar to *P. inflata*, whereas the others were close to *P. depauperata*. *Petunia bajeensis* displayed a pattern similar to that of *P. integrifolia* (Fig. 4c). For K = 5, each taxon presented a different genetic constitution, with most of the *P. bajeensis* and *P. integrifolia* individuals sharing the same genetic component, while *P. depauperata*, *P. inflata*, and *P. interior* were characterised by different predominant genetic components (Fig. 4d).

The phylogenetic tree based on all three AFLPs had well-supported branches, with the majority of the groups related to taxon morphology (Fig. 3b). The first clade was formed by most of the *P. bajeensis* individuals (PP = 1.0), while the second grouped individuals of all the other species of the *P. integrifolia* complex (PP = 1.0). This second clade was divided into two subgroups: the first included all individuals of *P. interior* and a single individual each of *P. inflata* and *P. bajeensis* (PP = 1.0). The second subgroup comprised all remaining individuals and species (PP = 0.85) and was further subdivided into two fully-supported clades, one formed only by *P. inflata* and the other by *P. integrifolia* and *P. depauperata* (Fig. 3b).

### Spatial distribution

The replicated models for the taxa resulted in AUC values >0.919 (Additional file 11: Table S7). Maxent analyses suggested that the climate variables offering the best explanation for the geographic distribution varied between the different taxa of the *P. integrifolia* complex: the “mean temperature of the warmest quarter” made the greatest contribution to the model in *P. bajeensis* (61.2%) and *P. integrifolia* (28.3%)*,* the “annual precipitation” for *P. depauperata* (82%), “temperature seasonality” for *P. inflata* (47.1%), and the “mean temperature of the coldest quarter” for *P. interior* (70.3%).

**Fig. 5:**
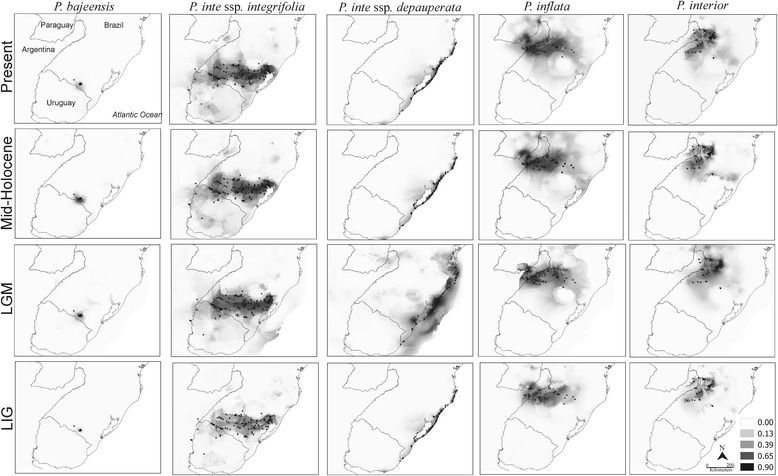
Ecological Niche Modelling for the *Petunia integrifolia* complex. Black dots represent the localities where species were found. Models were calibrated for the present, Mid-Holocene, Last Glacial Maximum (LGM) and Last Interglacial (LIG). Darker areas represent higher probabilities of occurrence. Country boundaries obtained from http://www.diva-gis.org

Ecological niche modelling over time (Fig. 5) showed that present and LIG climate conditions were even less suitable for *P. bajeensis* than MH and LMG conditions. The suitable areas for *P. integrifolia* were spread out over regions close to the Atlantic coast during the MH, and the modelling provided little evidence for the presence of this taxon in the coastal area under LGM and LIG conditions. The suitable areas for *P. depauperata* were larger under LGM conditions, when sea levels were lower, than under LIG, MH or present conditions. For *P. inflata,* the results of ENM were, for the most part, stable over LGM, MH, and present periods, but the suitable area was smallest in the LIG. The suitable area for *P. interior* was greater in size than in the present versus past-tested intervals except for the LIG. The stable areas for all taxa were very similar under LGM, MH, and present conditions, with the exception of *P. depauperata*, for which the suitable area has diminished in size since the LGM (Fig. 5). In general, suitable areas were smaller during the LIG for all taxa than during the other periods.

The analysis of biogeographical regions found *P. integrifolia*, *P. depauperata,* and *P. inflata* to be present in Bioregion 1; individuals of *P. integrifolia*, *P. inflata,* and *P. interior* were found in Bioregion 2; while in Bioregion 3, only *P. integrifolia* and *P. bajeensis* were found (Fig. 1a; Additional file 12: Table S8). The bioregions identified for all taxa of the *P. integrifolia* complex revealed that, while restricted to the Pampas region, *P. integrifolia* is present in all areas, whereas *P. inflata* and *P. interior* are found in the grasslands at higher elevations, in a region within the *Araucaria* forest domain. The reconstruction of the ancestral distribution (Fig. 1b) fitted the distribution of *P. integrifolia* and included the entire complex. All identified bioregions encompassed the suitable areas for each taxon that remained stable over time according to ENM results.

## Discussion

The *P. integrifolia* complex inhabits a transitional area [16]. Transitional areas are usually characterised by the presence of heterogeneous habitats; in the present case, three bioregions are found within a small geographical area (Fig. 1). Environmental heterogeneity is considered a trigger for speciation processes; here, speciation may have occurred without promoting deep morphological changes (Additional file 3: Box S2). On the other hand, phenotypic plasticity and ecological or geographical adaptations to a heterogeneous environment (luminosity, open or forested areas, and different types of soil) might explain some of the morphological variation among the populations of these closely related taxa of recent evolutionary origin.

Here, we compared the genetic diversity of five taxa of the *P. integrifolia* complex based on different molecular markers (sequences of plastid gene spacers, nuclear microsatellites, and AFLP profiles). We focused on phylogenetic context, group cohesiveness, and niche conservatism, considering multiple individuals and populations per taxon. We evaluated the ability of the different markers to correctly assign individuals to their corresponding morphological-based taxonomic group and modelled niche distribution in an effort to identify the drivers of diversification and taxon delimitation. Nevertheless, the different molecular markers analysed in this study demonstrated differences in their potential to correctly assign individuals to their respective morphological taxa.

A recurrent pattern among *Petunia* species and subspecies is the absence of reciprocal monophyly and a large degree of interspecific plastid haplotype sharing, even between species that form morphologically well-delimited taxa [26, 29, 30]. Despite the minimal morphological differentiation within the *P. integrifolia* complex (Additional file 3: Box S2), plastid marker analyses confirmed the separation of *P. integrifolia*, *P. depauperata,* and *P. bajeensis* (Fig. 2) with a minimum of ancestral sharing.

Moreover, the *P. integrifolia* subspecies (*P. integrifolia* and *P. depauperata*) are equally distant from each other as *P. integrifolia* and *P. bajeensis* (Figs. 2 and 3). Based on this, we suggest considering *P. integrifolia* and *P. depauperata* as two independent species, which can be identified based on their habit (procumbent in *P. depauperata* and decumbent in *P. integrifolia*) as well as the shape of their leaves and their geographical distribution in nature (Additional file 3: Box S2). The flowers of *P. depauperata* are significantly smaller than those of *P. integrifolia* [35], and the corolla is darker in *P. depauperata*. Changes in nomenclature are necessary and shall be discussed in an appropriate forum.

For *P. interior* and *P. inflata*, cpDNA analysis revealed a more complex pattern*.* Four *P. interior* populations from the northernmost region of the species’ distribution, where *P. inflata* does not occur, and two populations from its southernmost area, which are near populations of *P. inflata* (see Fig. 1), form a separate group. However, the majority of populations of both species are grouped together (Figs. 2 and 3). In accordance with the results of the Bayesian Skyline Plot (Additional file 7: Figure S2) and niche modelling (Fig. 5), this pattern could be the result of a recent population expansion of *P. interior* to the south. However, because haplotype sharing among the populations is rare, recent hybridization does not offer the best explanation for this pattern. Haplotype sharing has been described as a consequence of recent interspecific hybridization of *P. axillaris* and *P. exserta* [15, 26]; however, in that case, it was paired with various morphological signals. Other authors have found plastid haplotype sharing in the absence of hybridization, a finding that likely reflects differing patterns of genetic drift between recently diverged species [21]. Such incomplete lineage sorting supports the idea that insufficient time has elapsed to allow complete divergence of *Petunia* plastids [27]. The most recent common ancestor (MRCA) of the *Petunia* genus is thought to have lived a minimum of 1.3 Ma ago (Mya) [21] and a maximum of 2.8 Mya [83]. The divergence of *P. integrifolia* and *P. depauperata* has been estimated at 400 Kya [27], and in the present work, group II is estimated to have diverged around 687 Kya, while *P. bajeensis* diverged from the other taxa ~199 Kya, making incomplete lineage sorting a realistic possibility [84–86]. Alternatively, these results can be interpreted as an indication that genetically, these individuals belong to the same species, and that morphological differences may be attributed to phenotypic plasticity. We thus suggest a taxonomic review.

Microsatellites are highly variable and can be highly effective in discriminating closely related species [87]. On the other hand, even though AFLPs generally have lower mutation rates than SSRs, studies in ferns [88], mangroves [89], and *Quercus* [90] have shown that AFLPs are more efficient at distinguishing populations than microsatellites. To date, there have been few studies comparing the effectiveness of microsatellites and AFLPs in discriminating plant species. It has been suggested that, in animals, both types of markers have an approximately 70% chance to correctly discriminate between species and assign individuals to their respective groups [91]. In the present study, we found SSRs and AFLPs (Figs. 3 and 4) less effective than cpDNA haplotypes (Fig. 2) in discriminating between the species of the *P. integrifolia* complex, according to the currently accepted morphological classification. The genetic structure revealed by the AMOVA highlights this difference among markers (Table 2): whereas 64.4% of the total cpDNA variation was between groups, the corresponding variation was only 38.6% for SSRs and 17.2% for AFLPs. In addition, we believe that the high variability observed within taxa could explain the discrepancies found between the AFLP-based tree and Structure analyses and the results obtained from these analyses using the other markers (Figs. 3 and 4).

The results of the Structure analyses using AFLPs and SSRs agree with the complicated morphological classification of the *P. integrifolia* complex and are in line with the phenotypic continuum found for a range of morphological traits (Additional file 1: Figure S1 and Additional file 3: Box S2). Plastid markers usually show more local structure, mostly because their maternal inheritance reflects the importance of the habitat in lineage isolation [29, 92].

It is important to highlight that the geographical distribution of *P. bajeensis* falls completely within the range of *P. integrifolia*. However, there was no suggestion of population reduction (Additional file 7: Figure S2) or hybridization (based on cpDNA and SSRs analyses) between *P. bajeensis* and *P. integrifolia*, and the populations of *P. bajeensis* occupy a different bioregion, where some populations of *P. integrifolia* also occur. The ecological and/or environmental factors responsible for this isolation remain to be determined. Despite morphological similarities, phylogenetic analyses [24, 25] found that *P. bajeensis* is not closely related to the remaining taxa of the complex; it is the sister species of the highland subclade in the short corolla tube clade.

The *P. integrifolia* complex is comprised of four geographically isolated and stable lineages (Fig. 5), which are associated to different bioregions (Fig. 1). The fact that these closely related taxa occupy very different habitats, such as the coastal plains, the Pampas, and the Atlantic forest, suggests that eco-geographical isolation has acted as one of the most significant reproductive barriers within the genus *Petunia*. The dynamic of new adaptations spreading through a population might be influenced by the levels of gene flow and demographic structure [93, 94]. The phylogeographic patterns found in the *P. integrifolia* complex could therefore be the result of low levels of gene flow through seed dispersion, either by free fall or barochory [16, 95], combined with differential selection in a heterogeneous environment. This is consistent with the fact that these species share a pollinator, and with the absence of reproductive barriers, at least in controlled situations [16]. Up to date, there are no studies on the pollination biology of these species, and the available data come from sporadic field observations.

The geographical area inhabited by the taxa of the *P. integrifolia* complex is of a variable topography and is characterised by a humid subtropical climate (Cfa) [96]. It is a region of transition between two important Neotropical phytogeographical formations: the Pampas grassland [97] and the Brazilian Atlantic Forest (BAF) [98]. In the central portion of Rio Grande do Sul, for example, populations of *P. integrifolia* and *P. inflata* are located only ca. 22 km from one another, but while *P. integrifolia* inhabits the Pampas grasslands up to a maximum elevation of 167 m, *P. inflata* and *P. interior* are found in mosaics of grasslands and BAF above 450 m, in the *Araucaria* forest domain. Both the Pampas grassland and the BAF have undergone a series of changes in range and floristic composition during the Quaternary [99]. Even during warm periods, open fields naturally covered the Pampas grasslands with small patches or long strips of gallery forests along rivers and on the escarpments of low-elevation sandstone hills [100]. In the southern BAF, typified by mosaics of high-elevation grasslands and forests, the dynamics of the Quaternary are also well documented through pollen records [100, 101]. During glacial periods, forests were restricted to more humid areas along watercourses and slopes, enabling the expansion of the grassland areas. In contrast, the conditions of the interglacial periods allowed forests to gain more territory over the grasslands [99]. The plant migrations triggered by the Quaternary climate oscillations, combined with the non-uniform landscape composition, may have contributed to the diversification of the evolutionary lineages of the *P. integrifolia* complex. This dynamic also underlies the diversification and range expansion of other *Petunia* species [21] and of the genus as a whole [24], and mirrors that of other plant species from the same region [31, 102, 103], in line with the theory of Andean components in the southern Brazilian flora [104]. The differentiation between habitats and the stability found in niche modelling analysis reinforce the idea that there are four genetic lineages: *P. integrifolia*, *P. depauperata*, *P. bajeensis,* and *P. inflata* + *P. interior*.

## Conclusions

The species is one of the fundamental units of biology. In nature, plant species identification is often complicated by the presence of a continuum of morphological traits across taxa. Overall, our results suggest that the *Petunia integrifolia* species complex represents four historically isolated lineages. We suggest *P. depauperata* be elevated to species level and *P. inflata* and *P. interior* status should be investigated in a broader approach including morphological data. We also point out *P. bajeensis* is not part of the complex because its phylogenetic position, which could be attributed to incomplete lineage sorting. The cpDNA markers were more efficient to discriminate among *Petunia* taxa classified based on morphological traits than SSRs and AFLPs. The Niche modelling and bioregions analyses revealed that the habitat played an important role in preserving species boundaries over time. The sharing of morphological, ecological, and genetic characters do not necessarily accommodate a definition according to the biological species concept, but are completely in agreement with the definition of a species as a metapopulation. Beyond the ornamental *P. hybrida*, a popular model of molecular and developmental genetics, wild *Petunia* species thus serve as a remarkable case study of evolutionary biology.

## Additional files

Additional file 1: Figure S1. Morphologies of the species of the *Petunia integrifolia* complex and evolutionary relationships among *Petunia* species. (A) *P. bajeensis;* (B) *P. integrifolia* Ss*p. depauperata;* (C) *P. inflata*; (D) *P. integrifolia* Ss*p. integrifolia;* (E) *P. interior*; (F) Phylogenetic tree adapted from Reck-Kortmann et al. (2014): the long corolla tube clade is shown in pink, the short corolla tube clade is shown in black; species of the *P. integrifolia* complex are in bold type. (PNG 4747 kb)

Additional file 2: Box S1. Simplified taxonomic changes of the *Petunia integrifolia* complex over time. (DOCX 19 kb)

Additional file 3: Box S2. Morphological characterisation of the *Petunia integrifolia* complex. (DOCX 14 kb)

Additional file 4: Table S1. Sample Collection. (DOCX 111 kb)

Additional file 5: Table S2. Collection sites used in the Environmental Niche Modelling (ENM) analysis to the taxa of *Petunia integrifolia* complex. (DOCX 47 kb)

Additional file 6: Table S3. Plastid haplotypes observed for each species of the *Petunia integrifolia* complex. (DOCX 13 kb)

Additional file 7: Figure S2. Changes in the effective population size over time (years ago) for each species of the *Petunia integrifolia* complex. Bayesian skyline plot showing the effective population size fluctuation throughout time for *P. bajeensis*, *P. integrifolia* ssp.*, integrifolia*, *P. inflata* and *P. interior* (solid line, median estimators of Ne; blue lines, confidence interval). (TIFF 70 kb)

Additional file 8: Table S4. Diversity indices for the *Petunia integrifolia* complex based on microsatellite variation. (DOCX 17 kb)

Additional file 9: Table S5. Microsatellite-based diversity indices for species of *Petunia integrifolia* complex. (DOCX 14 kb)

Additional file 10: Table S6. AFLP-based genetic diversity of the *Petunia integrifolia* complex. (DOCX 12 kb)

Additional file 11: Table S7. AUC values and standard deviations of the Ecological Niche Modeling. (DOCX 14 kb)

Additional file 12: Table S8. Selected bioregions for each species of the *Petunia integrifolia* complex. (DOCX 12 kb)

## Abbreviations

#P_loc: AFLP polymorphic loci
A: Allele number
AFLP: Amplified fragment length polymorphism
AIC: Akaike information criterion
AMOVA: Analysis of molecular variance
AR: Allele richness
AUC: Area under the curve
BAF: Brazilian Atlantic Forest
BHCB: Universidade Federal de Minas Gerais Herbarium
BI: Bayesian inference
bp: Base pair
BSP: Bayesian Skyline Plot
Cfa: Humid subtropical climate
cpDNA: Plastid DNA
CTAB: Cetyltrimethylammonium bromide
ENM: Ecological niche modelling
ESS: Effective sample sizes
EX: Exclusive alleles
F_*IS*_: Inbreeding coefficient
GD: Gene diversity
GPS: Global positioning system
GTR: General Time Reversible
h: Haplotypic diversity
*HE*: Expected heterozygosity
Hj: AFLP Nei’s gene diversity
HKY: Hasegawa, Kishino, and Yano nucleotide substitution model
*HO*: Observed heterozygosity
ICN: Universidade Federal do Rio Grande do Sul Herbarium
indels: Insertions/deletions
K: Groups tested in STRUCTURE software
Kya: Thousand years ago
LGM: Last Glacial Maximum
LIG: Last Interglacial
MCMC: Markov chain Monte Carlo
MH: Mid-Holocene
MRCA: Most recent common ancestor
Mya: Million years ago
NUL: Proportion of null alleles
PCR: Polymerase Chain Reaction
PEG: Polyethylene glycol
PIC: Polymorphic information content
PP: Posterior probability
R_*ST*_: F-statistic analogues
SSR: Simple sequence repeats
π: Nucleotide diversity

## References

[1] Blair C, Méndez de la Cruz FR, Law C, Murphy RW (2015). Molecular phylogenetics and species delimitation of leaf-toed geckos (Phyllodactylidae: *Phyllodactylus*) throughout the Mexican tropical dry forest. Mol Phylogenet Evol.

[2] Leaché AD, Fujita MK (2010). Bayesian species delimitation in west African forest geckos (*Hemidactylus fasciatus*). Proc R Soc Lond B Biol Sci.

[3] Nicola MV, Sede SM, Pozner R, Johnson LA (2014). Phylogeography and palaeodistribution modelling of *Nassauvia* subgenus *Strongyloma* (Asteraceae): exploring phylogeographical scenarios in the Patagonian steppe. Ecology and Evolution.

[4] Sites Jr JW, Marshall JC (2004). Operational criteria for delimiting species. Annu Rev Ecol Evol Syst.

[5] Strasburg JL, Rieseberg LH (2011). Interpreting the estimated timing of migration events between hybridizing species. Mol Ecol.

[6] Sukumaran J, Knowles LL (2017). Multispecies coalescent delimits structure, not species. PNAS.

[7] Yeung AKL, Tsai PW, Chesser RT, Lin RC, Yao CT, Tian XH, Li SH (2011). Testing founder effect speciation: divergence population genetics of the spoonbills *Platalea regia* and *Pl. Minor* (Threskiornithidae, Aves). Mol Biol Evol.

[8] Hart MW (2011). The species concept as an emergent property of population biology. Evolution.

[9] Givnish TJ, Evans TM, Zjhra ML, Patterson TB, Berry PE, Sytsma KJ (2000). Molecular evolution, adaptive radiation, and geographic diversification in the amphi-Atlantic family Rapateaceae: evidence from *ndhF* sequences and morphology. Evolution.

[10] Abbott RJ (2007). Comes HP. Blowin' in the wind: the transition from ecotype to species. New Phytol.

[11] van der Niet T, Johnson SD (2012). Phylogenetic evidence for pollinator-driven diversification of angiosperms. Trends Ecol Evol.

[12] Schäffler I, Balao F, Dötterl S (2012). Floral and vegetative cues in oil-secreting and non-oil-secreting *Lysimachia* species. Ann Bot.

[13] Sun M, Gross K, Schiestl FP (2014). Floral adaptation to local pollinator guilds in a terrestrial orchid. Ann Bot.

[14] Vandenbussche M, Chambrier P, Rodrigues-Bento S, Morel P (2016). *Petunia*, your next supermodel?. Front Plant Sci.

[15] Segatto ALA, Ramos-Fregonezi AMC, Bonatto SL, Freitas LB (2014). Molecular insights into the purple–flowered ancestor of garden petunias. Am J Bot.

[16] Stehmann JR, Lorenz-Lemke AP, Freitas LB, Semir J, Gerats T, Strommer J (2009). The genus *Petunia*. *Petunia*: evolutionary, developmental and physiological genetics.

[17] Tsukamoto T, Ando T, Kokubun H, Watanabe H, Tanaka R, Hashimoto G, Marchesi E, Kao T (1998). Differentiation in the status of self-incompatibility among all natural taxa of *Petunia* (Solanaceae). Acta Phytotaxonomica Geobotanica.

[18] Smith TB, Kark S, Schneider CJ, Wayne RK, Moritz C (2001). Biodiversity hotspots and beyond: the need for conserving environmental transitions. Trends Ecol Evol.

[19] Ando T, Kokubun H, Watanabe H, Tanaka N, Yukawa T, Hashimoto G, Marchesi E, Suárez E, Basualdo IL (2005). Phylogenetic analysis of *Petunia* Sensu Jussieu (Solanaceae) using chloroplast DNA RFLP. Ann Bot.

[20] Kulcheski FR, Muschner VC, Lorenz-Lemke AP, Stehmann JR, Salzano FM, Bonatto SL, Freitas LB (2006). Molecular phylogenetic analysis of *Petunia* Juss. (Solanaceae). Genetica.

[21] Lorenz-Lemke AP, Togni PD, Mäder G, Kriedt RA, Stehmann JR, Salzano FM, Bonatto SL, Freitas LB (2010). Diversification of plant species in a subtropical region of eastern south American highlands: a phylogeographic perspective on native *Petunia* (Solanaceae). Mol Ecol.

[22] Chen S, Matsubara K, Omori T, Kokubun H, Kodama H, Watanabe H, Hashimoto G, Marchesi E, Bullrich L, Ando T (2007). Phylogenetic analysis of the genus *Petunia* (Solanaceae) based on the sequence of the Hf1 gene. J Plant Res.

[23] Kriedt RA, Cruz GMQ, Bonatto SL, Freitas LB (2014). Novel transposable elements in Solanaceae: evolutionary relationships among *Tnt1*-related sequences in wild *Petunia* species. Plant Mol Biol Report.

[24] Reck-Kortmann M, Silva-Arias GA, Segatto ALA, Mäder G, Bonatto SL, Freitas LB (2014). Multilocus phylogeny reconstruction: new insights into the evolutionary history of the genus *Petunia*. Mol Phylogenet Evol.

[25] Segatto ALA, Thompson CE, Freitas LB (2016). Contribution of *WUSCHEL*-related homeobox (*WOX*) genes to identify the phylogenetic relationships among *Petunia* species. Genet Mol Biol.

[26] Lorenz-Lemke AP, Mäder G, Muschner VC, Stehmann JR, Bonatto SL, Salzano FM, Freitas LB (2006). Diversity and natural hybridization in a highly endemic species of *Petunia* (Solanaceae): a molecular and ecological analysis. Mol Ecol.

[27] Ramos-Fregonezi AMC, Fregonezi JN, Cybis GB, Fagundes NJR, Bonatto SL, Freitas LB (2015). Were sea level changes during the Pleistocene in the South Atlantic coastal plain a driver of speciation in *Petunia* (Solanaceae)?. BMC Evol Biol.

[28] Longo D, Lorenz-Lemke AP, Mäder G, Bonatto SL, Freitas LB (2014). Phylogeography of the *Petunia integrifolia* Complex in southern Brazil. Bot J Linn Soc.

[29] Segatto ALA, Cazé ALR, Turchetto C, Klahre U, Kuhlemeier C, Bonatto SL, Freitas LB (2014). Nuclear and plastid markers reveal the persistence of genetic identity: a new perspective on the evolutionary history of *Petunia exserta*. Mol Phylogenet Evol.

[30] Turchetto C, Fagundes NJR, Segatto ALA, Kuhlemeier C, Solís-Neffa VG, Speranza PR, Bonatto SL, Freitas LB (2014). Diversification in the south American pampas: the genetic and morphological variation of the widespread *Petunia axillaris* Complex (Solanaceae). Mol Ecol.

[31] Fregonezi JN, Turchetto C, Bonatto SL, Freitas LB (2013). Biogeographical history and diversification of *Petunia* and *Calibrachoa* (Solanaceae) in the Neotropical pampas grassland. Bot J Linn Soc.

[32] Watanabe H, Ando T, Iida S, Suzuki A, Buto K, Tsukamoto T, Hashimoto G, Marchesi E (1996). Cross compatibility of *Petunia* cultivars and *P. axillaris* with native taxa of *Petunia* in relation to their chromosome number. Journal of the Japanese Society for Horticultural Science.

[33] Turchetto C, Segatto ALA, Beduschi J, Bonatto SL, Freitas LB (2015). Genetic differentiation and hybrid identification using microsatellite markers in closely related wild species. AoB Plants.

[34] Ando T, Ishikawa N, Watanabe H, Kokubun H, Yanagisawa Y, Hashimoto G, Marchesi E, Suárez EA (2005). Morphological study of the *Petunia integrifolia* Complex (Solanaceae). Ann Bot.

[35] Ando T, Kurata M, Sasaki S, Ueda Y, Hashimoto G, Marchesi E (1995). Comparative morphological studies on intraspecific taxa of *Petunia integrifolia* (hook.) Schinz et Thell. (Solanaceae). Journal of Japanese Botany.

[36] Stehmann JR, Bohs L (2007). Nuevas combinaciones en Solanaceae. Darwin.

[37] Ando T, Hashimoto GA (1996). New Brazilian species of *Petunia* (Solanaceae) from interior Santa Catarina and Rio Grande do Sul, Brazil. Brittonia.

[38] Ando T, Hashimoto G (1998). Two new species of *Petunia* (Solanaceae) from southern Rio Grande do Sul, Brazil. Brittonia.

[39] Roy A, Frascaria N, MacKay J, Bousquet J (1992). Segregating random amplified polymorphic DNAs (RAPDs) in *Betula alleghaniensis*. Theor Appl Genet.

[40] Hamilton MB (1999). Four primers pairs for the amplification of chloroplast intergenic regions with intraspecific variation. Mol Ecol.

[41] Sang T, Crawford DJ, Stuessy TF, Chloroplast DNA (1997). Phylogeny, reticulate evolution, and biogeography of *Paeonia* (Paeoniaceae). Am J Bot.

[42] Dunn IS, Blattner FR (1987). Charons 36–40: multi-enzyme, high capacity, recombination deficient replacement vectors with polylinkers and polystuffers. Nucleic Acids Res.

[43] Tamura K, Stecher G, Peterson D, Filipski A, Kumar S (2013). MEGA6: molecular evolutionary genetics analysis version 6.0. Mol Biol Evol.

[44] Kelchner SA (2000). The evolution of non-coding chloroplast DNA and its application in plant systematics. Ann Mo Bot Gard.

[45] Kim KJ, Lee HL (2005). Widespread occurrence of small inversions in the chloroplast genomes of land plants. Molecules and Cells.

[46] Simmons MP, Ochoterena H (2000). Gaps as characters in sequence-based phylogenetic analyses. Syst Biol.

[47] Rozas J, Sánchez-Delbarrio JC, Messeguer X, Rozas R, DnaSP DNA (2003). Polymorphism analyses by the coalescent and other methods. Bioinformatics.

[48] Bandelt HJ, Forster P, Röhl A (1999). Median-joining networks for inferring intraspecific phylogenies. Mol Biol Evol.

[49] Expertise in software for genetic and engineering. http://www.fluxus-engineering.com. Accessed June 2017.

[50] Nei M (1987). Molecular evolutionary genetics.

[51] Excoffier L, Lischer HEL (2010). Arlequin suite ver 3.5: a new series of programs to perform population genetics analyses under Linux and windows. Molecular ecology. Resource.

[52] Drummond AJ, Suchard MA, Xie D, Rambaut A (2012). Bayesian phylogenetic with BEAUti and the BEAST 1.7. Mol Biol Evol.

[53] Darriba D, Taboada GL, Doallo R, Posada D (2012). JModelTest 2: more models, new heuristics and parallel computing. Nat Methods.

[54] Molecular Evolution, phylogenetics and epidemiology. http://tree.bio.ed.ac.uk/software/tracer/. Accessed June 2017.

[55] Molecular Evolution, phylogenetics and epidemiology. http://tree.bio.ed.ac.uk/software/figtree/. Accessed June 2017.

[56] Drummond AJ, Rambaut A, Shapiro B, Pybus OG (2005). Bayesian coalescent inference of past population dynamics from molecular sequences. Mol Biol Evol.

[57] Kriedt RA, Ramos-Fregonezi AMC, Beheregaray LB, Bonatto SL, Freitas LB (2011). Isolation, characterization, and cross-amplification of microsatellite markers for the *Petunia integrifolia* (Solanaceae) complex. Am J Bot.

[58] Goudet J (1995). FSTA Version 1.2: a computer program to calculate F-statistics. J Hered.

[59] Marshall TC, Slate J, Kruuk LEB, Pemberton JM (1998). Statistical confidence for likelihood-based paternity inference in natural populations. Mol Ecol.

[60] Pritchard JK, Stephens M, Donnelly P (2000). Inference of population structure using multilocus genotype data. Genetics.

[61] Falush D, Stephens MS, Pritchard JK (2003). Inference of population structure using multilocus genotype data: linked loci and correlated allele frequencies. Genetics.

[62] Evanno G, Regnaut S, Goudet J (2005). Detecting the number of clusters of individuals using the software STRUCTURE: a simulation study. Mol Ecol.

[63] Earl DA, von Holdt BM (2011). STRUCTURE HARVESTER: a website and program for visualizing STRUCTURE output and implementing the Evanno method. Conserv Genet Resour.

[64] Jakobsson M, Rosenberg NA (2007). CLUMPP: a cluster matching and permutation program for dealing with label switching and multimodality in analysis of population structure. Bioinformatics.

[65] Rosenberg NA (2004). DISTRUCT : a program for the graphical display of population structure. Mol Ecol Notes.

[66] Vos P, Hogers R, Bleeker M, Reijans M, van de Lee T, Hornes M, Kuiper M (1995). AFLP: a new technique for DNA fingerprinting. Nucleic Acids Res.

[67] Lynch M, Milligan B (1994). Analysis of population-genetic structure using RAPD markers. Mol Ecol.

[68] Vekemans X. AFLP-SURV version 1.0. Laboratoire de Génétique et Écologie Végétales. Université Libre de Bruxelles, Belgium. 2002; (distributed by the author).

[69] Ronquist F, Huelsenbeck JP (2003). MrBayes 3: Bayesian phylogenetic inference under mixed models. Bioinformatics.

[70] Felsenstein J (1981). Evolutionary trees from DNA sequences: a maximum likelihood approach. J Mol Evol.

[71] Species Link. http://www.splink.org.br. Accessed June 2017.

[72] Global Biodiversity Information Facility. http://www.gbif.org. Accessed June 2017.

[73] WorldClim – Global Climate Change. http://www.worldclim.org/. Accessed June 2017.

[74] Phillips SJ, Anderson RP, Schapire RE (2006). Maximum entropy modelling of species geographic distributions. Ecol Model.

[75] Hijmans RJ, van Etten J (2010). RASTER: package for reading, writing, and manipulating raster (grid) type geographic (spatial) data.

[76] R Development Core Team. R: A language and environment for statistical computing. Vienna: R foundation for statistical Computing 2010. http://www.R-project.org. Accessed June 2017.

[77] Peterson AT (2007). Why not, why where: the need for more complex models of simpler environmental spaces. Ecol Model.

[78] Nakazato T, Warren DL, Moyle LC (2010). Ecological and geographic modes of species divergence in wild tomatoes. Am J Bot.

[79] Edler D, Guedes T, Ziska A, Rosvall M, Antonelli A (2017). Infomap bioregions: interactive mapping of biogeographical regions from species distributions. Syst Biol.

[80] Vilhena DA, Antonelli A (2015). A network approach for identifying and delimiting biogeographical regions. Nat Commun.

[81] Infomap Biorregions. http://bioregions.mapequation.org. Accessed June 2017.

[82] Fitch WM (1971). Toward defining the course of evolution: minimum change for a specific tree topology. Syst Biol.

[83] Särkinen T, Bohs L, Olmstead RG, Knapp S (2013). A phylogenetic framework for evolutionary study of the nightshades (Solanaceae): a dated 1000-tip tree. BMC Evol Biol.

[84] Knowles LL (2000). Tests of Pleistocene speciation in montane grasshoppers (genus *Melanoplus*) from the sky islands of western North America. Evolution.

[85] Knowles LL (2001). Did the Pleistocene glaciations promote divergence? Tests of explicit refugial models in montane grasshoppers. Mol Ecol.

[86] Knowles LL, Carstens BC (2007). Delimiting species without monophyletic gene trees. Syst Biol.

[87] Duminil J, Kenfack D, Viscosi V, Grumiau L, Hardy OJ (2012). Testing species delimitation in sympatric species complexes: the case of an African tropical tree, *Carapa* spp. (Meliaceae). Mol Phylogenet Evol.

[88] Woodhead M, Russell J, Squirrell J, Hollingsworth PM, Mackenzie K, Gibby M, Powell W (2005). Comparative analysis of population genetic structure in *Athyrium distentifolium* (Pteridophyta) using AFLPs and SSRs from anonymous and transcribed gene regions. Mol Ecol.

[89] Maguire TL, Peakall R, Saenger P (2002). Comparative analysis of genetic diversity in the mangrove species *Avicennia marina* (Forsk.) Vierh. (Avicenniaceae) detected by AFLPs and SSRs. Theor Appl Genet.

[90] Mariette S, Cottrell J, Csaikl UM, Goikoechea P, Konig A, Lowe AJ, Van Dam BC, Barreneche T, Bodenes C, Streiff R, Burg K, Groppe K, Munro RC, Tabbener H, Kremer A (2002). Comparison of levels of genetic diversity detected with AFLP and microsatellite markers within and among mixed *Q. petraea* (Matt.) Liebl. And *Q. robur* L. stands. Silvae Genetica.

[91] Dupuis JR, Roe AD, Sperling FAH (2012). Multi-locus species delimitation in closely related animals and fungi: one marker is not enough. Mol Ecol.

[92] Derepas A, Dulieu H (1992). Inheritance of the capacity to transfer plastids by pollen parent in *Petunia hybrida* Hort. J Hered.

[93] Rieseberg LH, Burke JM (2001). The biological reality of species: gene flow, selection, and collective evolution. Taxon.

[94] Morjan CL, Rieseberg LH (2004). How species evolve collectively: implications of gene flow and selection for the spread of advantageous alleles. Mol Ecol.

[95] van der Pijl L. Principles of dispersal in higher plants. Berlin: Springer-Verlag. 1982. 3^rd^ ed. 214 p.

[96] Köppen W. Climatologia: com um estúdio de los climas de la tierra. In: Climatology. New Jersey: Laboratory of Climatology. 1948. 478 p.

[97] Cabrera AL, Willink A. Biogeografia de America Latina. Washington: Secretaria General de la Organización de los Estados Americanos. 1980. pp. 1-120.

[98] Martins FM (2011). Historical biogeography of the Brazilian Atlantic forest and the Carnaval-Moritz model of Pleistocene refugia: what do phylogeographical studies tell us?. Biol J Linn Soc.

[99] Behling H (2002). South and southeast Brazilian grasslands during late quaternary times: a synthesis. Palaeogeogr Palaeoclimatol Palaeoecol.

[100] Behling H, Pillar VP, Bauermann SG (2005). Late quaternary grassland (Campos), gallery forest, fire and climate dynamics, studied by pollen, charcoal and multivariate analysis of the São Francisco de Assis core in western Rio Grande do Sul (southern Brazil). Rev Palaeobot Palynol.

[101] Ledru MP, Salatino MLF, Ceccantini G, Salatino A, Pinheiro F, Pintaud JC (2007). Regional assessment of the impact of climatic change on the distribution of a tropical conifer in the lowlands of South America. Divers Distrib.

[102] Thode VA, Silva-Arias GA, Turchetto C, Segatto ALA, Mäder G, Bonatto SL, Freitas LB (2014). Genetic diversity and ecological niche modelling of the restricted *Recordia reitzii* (Verbenaceae) from southern Brazilian Atlantic forest. Bot J Linn Soc.

[103] Teixeira MC, Mäder G, Silva-Arias GA, Bonatto SL, Freitas LB (2016). Effects of past climate on *Passiflora actinia* (Passifloraceae) populations and insights into future species management in the Brazilian Atlantic forest. Bot J Linn Soc.

[104] Rambo B (1961). Migration routes of the south Brazilian rain forest. Pesquisas Botanica.

