# Supplementary figures and images for: Multiple markers, niche modelling, and bioregions analyses to evaluate the genetic diversity of a plant species complex
Source: BMC Evol Biol. 2017 Nov 29;17:234. doi: 10.1186/s12862-017-1084-y (PMC5707870; doi:10.1186/s12862-017-1084-y)

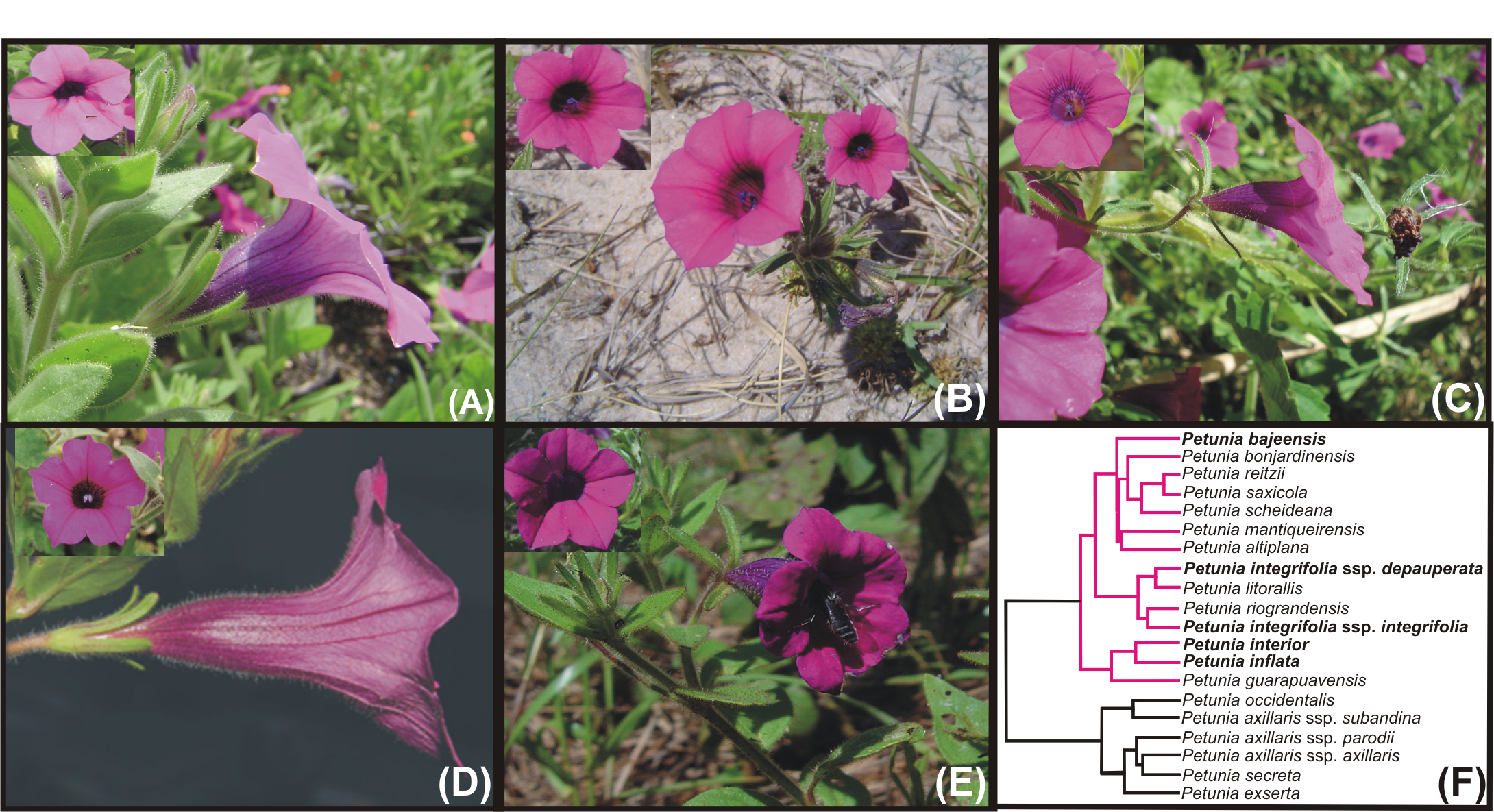

Supplement: Supplementary file 1 — Morphologies of the species of the Petunia integrifolia complex and evolutionary relationships among Petunia species. (A) P. bajeensis; (B) P. integrifolia Ssp. depauperata; (C) P. inflata; (D) P. integrifolia Ssp. integrifolia; (E) P. interior; (F) Phylogenetic tree adapted from Reck-Kortmann et al. (2014): the long corolla tube clade is shown in pink, the short corolla tube clade is shown in black; species of the P. integrifolia complex are in bold type. (PNG 4747 kb) [file 12862_2017_1084_MOESM1_ESM.png]

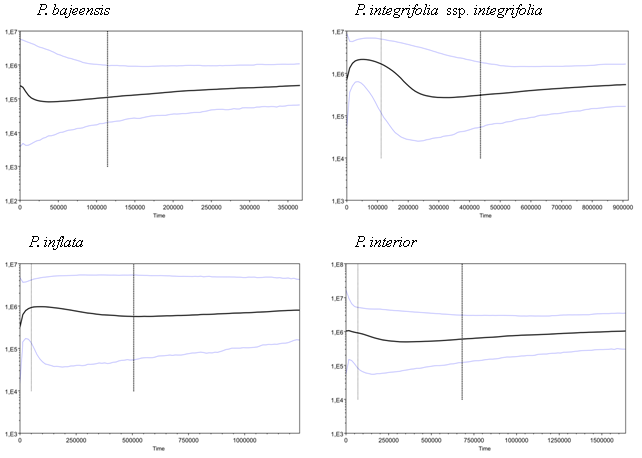

Supplement: Supplementary file 7 — Changes in the effective population size over time (years ago) for each species of the Petunia integrifolia complex. Bayesian skyline plot showing the effective population size fluctuation throughout time for P. bajeensis, P. integrifolia ssp., integrifolia, P. inflata and P. interior (solid line, median estimators of Ne; blue lines, confidence interval). (TIFF 70 kb) [file 12862_2017_1084_MOESM7_ESM.tif]
