# Supplementary material for: Multiple markers, niche modelling, and bioregions analyses to evaluate the genetic diversity of a plant species complex
Source: BMC Evol Biol. 2017 Nov 29;17:234. doi: 10.1186/s12862-017-1084-y (PMC5707870; doi:10.1186/s12862-017-1084-y)
Supplement: Supplementary file 2 — Simplified taxonomic changes of the Petunia integrifolia complex over time. (DOCX 19 kb) [file 12862_2017_1084_MOESM2_ESM.docx]

**Additional file 2: Box S1 -** Simplified taxonomic changes of the *Petunia integrifolia* complex over time

|  | Fries (1911) [105] | Schinz & Tellung (1915) [106] | Smith & Downs (1966) [107] | Wijsman (1982) [108] | Ando & Hashimoto (1993, 1996, 1998) [109,37,38] | Ando *et al.*  (2005)* [34] | Stehmann & Bohs (2007) [36] | Stehmann *et al.* (2009) [16] |
| --- | --- | --- | --- | --- | --- | --- | --- | --- |
| ***Salpinglosis integrifolia***  (Hooker, 1831) [110] | ***S. integrifolia*** | ***P. integrifolia*** | ***P. integrifolia*** | ***P. integrifolia* ssp. *integrifolia*** | ***P. riograndensis*** | ***P. riograndensis*** | ***P. riograndensis*** | ***P. integrifolia* ssp*. integrifolia*** |
|  |  |  |  |  | ***P. integrifolia*** | ***P. integrifolia*** | ***P. integrifolia*** |  |
| ***Petunia violaceae*** (Lindley, 1834) [111] | ***P. violaceae*** |  |  |  |  |  |  |  |
| ***Petunia dichotoma*** (Sendtn, 1846) [112] | ***P. dichotoma*** |  | ***P. integrifolia* ssp. *depauperata*** | ***P. integrifolia* ssp. *depauperata*** | ***P. integrifolia* ssp. *depauperata*** | ***P. integrifolia* ssp. *depauperata*** | ***P. integrifolia* ssp*. depauperata*** | ***P. integrifolia* ssp. *depauperata*** |
|  |  |  | ***P. littoralis*** | ***P. littoralis*** | ***P. littoralis*** | ***P. littoralis*** |  |  |
|  | ***P. inflata*** | ***P. inflata*** | ***P. inflata = P. integrifolia*** | ***P. integrifolia* ssp*. inflata*** | ***P. inflata*** | ***P. inflata*** | ***P. inflata*** | ***P. inflata*** |
|  |  |  |  |  | ***P. interior*** | ***P. interior*** | ***P. interior*** | ***P. interior*** |
|  |  |  |  |  | ***P. bajeensis*** | ***P. bajeensis*** | ***P. bajeensis*** | ***P. bajeensis*** |

* Complex proposition and description; in black, names proposed for the first time; in grey, non-revised names.
