# Supplementary material for: Multiple markers, niche modelling, and bioregions analyses to evaluate the genetic diversity of a plant species complex
Source: BMC Evol Biol. 2017 Nov 29;17:234. doi: 10.1186/s12862-017-1084-y (PMC5707870; doi:10.1186/s12862-017-1084-y)
Supplement: Supplementary file 3 — Morphological characterisation of the Petunia integrifolia complex. (DOCX 14 kb) [file 12862_2017_1084_MOESM3_ESM.docx]

**Additional file 3: Box S2 -** Morphological characterization of the *Petunia integrifolia* species complex

| Species | *P. bajeensis* | *P. integrifolia* ssp*. integrifolia* | *P. integrifolia* ssp*. depauperata* | *P. inflata* | *P. interior* |
| --- | --- | --- | --- | --- | --- |
| Habit | Decumbent | Decumbent | Procumbent | Ascendant | Erect |
| Leaves | Ovate, elliptic or oblong | Elliptic or obovate | Linear | NR | Oblong to broadly linear |
| Stems and branches | Foliose stems, densely glandular and pilose | Stem divided in two branches | Long and prostrated stems | NR | Stem divided in three branches |
| Calyx | Large, divided in 5 segments | Out-curved lobes | Deeply lobed | Unfolded and straight lobes | Strongly recurved lobes |
| Corolla | Funnelform, with intrude throat | Funnelform, with flat throat | Funnelform, small | Slightly constricted tube | Funnelform |
| Flower colour | Purple | Reddish-purple | Dark purple | Bright reddish-purple | Pale purple with deep purple strips |
| Filaments and stamens | Adnate more than 7 mm to the base of corolla tube | Adnate 2-5 mm to the base of corolla tube | NR | NR | Incurved in the upper portion; long stamens making contact with each other |
| Anthers | Flat and introrse | Flat and reverse | NR | NR | Channelled lobes |
| Stigma | Below the anthers of the longest pair of stamens | Between the anthers of long and medium stamens | NR | Between the anthers of long and medium stamens | Between the anthers of long and medium stamens |
| Fruit | 5 - 7 mm | < 8 mm | < 8 mm | > 9 mm | 5 - 6 mm |
| Pedicel | Deflexed | Deflexed | Deflexed | Inflexed | Weakly deflexed |
| Seeds | 0.5 - 0.7 mm | > 6 mm | > 6 mm | < 0.5 mm | 0.5 - 0.7 mm |
| Chromosome number | 2n=14 | 2n=14 | 2n=14 | 2n=14 | 2n=14 |
| Geographical distributions | Extreme southern region, Rio Grande do Sul Brazilian state (Pampas region) | Argentina, Uruguay, and Rio Grande do Sul Brazilian state (Pampas region) | Uruguay and Rio Grande do Sul and Santa Catarina Brazilian states (coastal region) | Argentina, Paraguay, and Rio Grande do Sul and Santa Catarina Brazilian states (Brazilian Plato), Argentina | Northern of Rio Grande do Sul and western of Santa Catarina Brazilian states (Brazilian Plato) |
| Environment | Along the roadside slopes and disturbed areas | Latosols and disturbed areas | Sandy soils along the coast | Altitudinal open fields in *Araucaria* forest | Altitudinal open fields in *Araucaria* forest |

Based on Ando and Hashimoto (1996; 1998) [37,38]; Ando *et al.* (2005) [34]; Stehmann and Bohs (2007) [36]; Stehmann *et al.* (2009) [16]; Wijsman and De Jong (1985) [113]; NR – no reference
