# Supplementary material for: Multiple markers, niche modelling, and bioregions analyses to evaluate the genetic diversity of a plant species complex
Source: BMC Evol Biol. 2017 Nov 29;17:234. doi: 10.1186/s12862-017-1084-y (PMC5707870; doi:10.1186/s12862-017-1084-y)
Supplement: Supplementary file 4 — Sample Collection. (DOCX 111 kb) [file 12862_2017_1084_MOESM4_ESM.docx]

**Additional file 4: Table S1** Sample information

| Taxon | Collection code | Individual code | SSR | AFLP | *trnH-psbA** | *trnS-trnG** | Geographic coordinates (UTM) | Voucher |
| --- | --- | --- | --- | --- | --- | --- | --- | --- |
| *Petunia bajeensis* T. Ando and Hashim. | B1 | baje02 | x |  | KX761613 | KX761710 | 31.4098S; 54.1349W | BHCB 102127 |
|  |  | baje03 | x |  | KX761613 | KX761710 |  |  |
|  |  | baje04 |  |  | KX761613 | KX761710 |  |  |
|  |  | baje05 | x |  | KX761613 | KX761710 |  |  |
|  |  | baje06 | x |  | KX761613 | KX761710 |  |  |
|  |  | baje07 |  |  | KX761613 | KX761710 |  |  |
|  |  | baje08 | x |  | KX761613 | KX761710 |  |  |
|  |  | baje09 | x |  | KX761613 | KX761710 |  |  |
|  |  | baje10 |  | x | KX761613 | KX761710 |  |  |
|  |  | baje11 | x |  | KX761613 | KX761710 |  |  |
|  |  | baje12 | x |  | KX761613 | KX761710 |  |  |
|  |  | baje13 |  |  | KX761613 | KX761710 |  |  |
|  |  | baje14 | x | x | KX761613 | KX761710 |  |  |
|  |  | baje15 | x | x | KX761613 | KX761710 |  |  |
|  |  | baje16 |  |  | KX761613 | KX761710 |  |  |
|  |  | baje18 | x | x | NA | NA |  |  |
|  |  | baje19 | x |  | KX761613 | KX761710 |  |  |
|  |  | baje20 | x | x | NA | NA |  |  |
|  | B2 | baj33 |  | x | KX761614 | KX761711 |  | BHCB156819 |
|  |  | baj34 |  |  | KX761615 | KX761712 |  |  |
|  |  | baj35 |  | x | KX761614 | KX761711 |  |  |
|  |  | baje36 |  |  | KX761614 | KX761711 |  |  |
|  |  | baje38 | x |  | KX761614 | KX761711 |  |  |
|  |  | baje39 | x | x | KX761614 | KX761711 |  |  |
|  |  | baje40 | x |  | KX761614 | KX761711 |  |  |
|  |  | baje41 | x | x | KX761614 | KX761711 |  |  |
|  |  | baje42 | x | x | KX761614 | KX761711 |  |  |
|  | B3 | inte103 |  |  | KX761698 | KX761795 |  | BHCB156819 |
| *Petunia integrifolia* ssp. *depauperata* R. E. Fr. | D1 | depa01 | x |  | KX761616 | KX761713 | 27.5229S; 48.4171W | BHCB 80104 |
|  |  | depa02 | x |  | KX761617 | KX761714 |  |  |
|  |  | depa03 | x |  | NA | NA |  |  |
|  |  | depa04 | x |  | NA | NA |  |  |
|  |  | depa05 | x |  | NA | NA |  |  |
|  |  | depa06 | x |  | KX761617 | KX761714 |  |  |
|  |  | depa07 | x |  | KX761617 | KX761714 |  |  |
|  |  | depa08 |  |  | KX761617 | KX761714 |  |  |
|  |  | depa09 |  | x | NA | NA |  |  |
|  |  | depa10 | x |  | NA | NA |  |  |
|  |  | depa11 | x |  | KX761616 | KX761713 |  |  |
|  |  | depa12 | x |  | NA | NA |  |  |
|  |  | depa13 | x |  | NA | NA |  |  |
|  |  | depa14 | x |  | KX761616 | KX761713 |  |  |
|  |  | depa25 |  |  | KX761616 | KX761713 |  |  |
|  |  | depa26 |  |  | KX761616 | KX761713 |  |  |
|  |  | depa29 |  |  | KX761616 | KX761713 |  |  |
|  |  | depa30 |  |  | KX761616 | KX761713 |  |  |
|  | D2 | depa31 |  |  | KX761618 | KX761715 | 30.3590S; 50.2931W | BHCB 99791 |
|  | D3 | depa38 |  |  | KX761618 | KX761715 | 30.2489S; 50.2314W | BHCB 99791 |
|  | D4 | depa62 |  |  | KX761618 | KX761715 | 30.2470; 50.2304W | BHCB 99791 |
|  | D5 | depa73 |  |  | KX761618 | KX761715 | 30.2617S; 50.2360W | BHCB 99791 |
|  |  | depa74 |  |  | KX761618 | KX761715 |  |  |
|  |  | depa75 |  |  | KX761618 | KX761715 |  |  |
|  |  | depa76 |  |  | KX761618 | KX761715 |  |  |
|  |  | depa77 |  |  | KX761618 | KX761715 |  |  |
|  |  | depa78 |  |  | KX761618 | KX761715 |  |  |
|  |  | depa79 |  |  | KX761618 | KX761715 |  |  |
|  |  | depa80 |  |  | KX761618 | KX761715 |  |  |
|  |  | depa81 |  |  | KX761618 | KF035646 |  |  |
|  |  | depa82 |  |  | KX761618 | KX761715 |  |  |
|  | D6 | depa117 |  |  | KX761618 | KX761715 | 30.3407S; 50.2679W | BHCB 99791 |
|  |  | depa118 |  |  | KX761618 | KX761715 |  |  |
|  |  | depa123 |  | x | KX761619 | KX761716 |  |  |
|  | D7 | depa131 |  |  | KX761618 | KX761715 | 30.1741S; 50.2015W | BHCB 99791 |
|  |  | depa133 |  |  | KX761618 | KX761715 |  |  |
|  |  | depa134 |  |  | KX761618 | KX761715 |  |  |
|  |  | depa136 |  |  | KX761618 | KX761715 |  |  |
|  |  | depa137 |  |  | KX761618 | KX761715 |  |  |
|  | D8 | depa158 |  |  | KX761618 | KX761715 | 30.1538S; 50.1942W | BHCB 87286 |
|  | D9 | depa177 |  |  | KX761620 | KX761717 | 30.0016S; 50.1307W | BHCB 87286 |
|  |  | depa184 |  |  | KX761620 | KX761717 |  |  |
|  |  | depa195 |  |  | KX761620 | KX761717 |  |  |
|  | D10 | depa197 |  |  | KX761621 | KX761718 | 29.8675S; 50.0687W | BHCB 87286 |
|  |  | depa198 |  |  | KX761621 | KX761718 |  |  |
|  |  | depa199 |  |  | KX761620 | KX761717 |  |  |
|  |  | depa200 |  |  | KX761621 | KX761718 |  |  |
|  | D11 | depa204 |  | x | KX761622 | KX761719 | 29.8589S; 50.0646W | BHCB 87286 |
|  | D12 | depa355 |  |  | KX761617 | KX761714 | 29.4319S; 49.7970W | BHCB 87286 |
|  |  | depa356 |  |  | KX761617 | KX761714 |  |  |
|  |  | depa357 |  |  | KX761617 | KX761714 |  |  |
|  |  | depa358 |  |  | KX761617 | KX761714 |  |  |
|  | D13 | depa449 |  |  | KX761620 | KX761717 | 29.8133S; 50.2429W | BHCB 104842 |
|  |  | depa456 |  |  | KX761623 | KX761720 |  |  |
|  |  | depa457 |  |  | KX761620 | KX761717 |  |  |
|  |  | depa481 |  |  | KX761620 | KX761717 |  |  |
|  |  | depa492 |  |  | KX761623 | KX761720 |  |  |
|  |  | depa501 |  |  | KX761620 | KX761717 |  |  |
|  |  | depa502 |  |  | KX761620 | KX761717 |  |  |
|  |  | depa503 |  |  | KX761620 | KX761717 |  |  |
|  |  | depa504 |  |  | KX761620 | KX761717 |  |  |
|  | D14 | depa505 |  |  | KX761623 | KX761720 | 29.8781S; 50.5132W | BHCB 87286 |
|  |  | depa506 |  |  | KX761623 | KX761720 |  |  |
|  |  | depa507 |  |  | KX761623 | KX761720 |  |  |
|  |  | depa508 |  |  | KX761624 | KX761721 |  |  |
|  |  | depa509 |  |  | KX761623 | KX761720 |  |  |
|  |  | depa510 |  |  | KX761624 | KX761721 |  |  |
|  | D15 | depa609 |  |  | KX761624 | KX761721 | 29.9052S; 50.4262W | BHCB 87286 |
|  |  | depa610 |  |  | KX761624 | KX761721 |  |  |
|  |  | depa611 |  |  | KX761620 | KX761717 |  |  |
|  |  | depa613 |  |  | KX761623 | KX761720 |  |  |
|  |  | depa614 |  |  | KX761623 | KX761720 |  |  |
|  |  | depa615 |  |  | KX761620 | KX761717 |  |  |
|  |  | depa616 |  |  | KX761620 | KX761717 |  |  |
|  |  | depa617 |  |  | KX761624 | KX761721 |  |  |
|  |  | depa618 |  |  | KX761624 | KX761721 |  |  |
|  | D16 | depa621 |  |  | KX761617 | KX761714 | 30.5409S; 50.4196W | BHCB 87286 |
|  |  | depa622 |  |  | KX761617 | KX761714 |  |  |
|  |  | depa623 |  |  | KX761617 | KX761714 |  |  |
|  |  | depa624 |  |  | KX761625 | KX761722 |  |  |
|  |  | depa625 |  |  | KX761617 | KX761714 |  |  |
|  |  | depa626 |  |  | KX761625 | KX761722 |  |  |
|  |  | depa627 |  |  | KX761617 | KX761714 |  |  |
|  |  | depa629 |  |  | KX761625 | KX761722 |  |  |
|  |  | depa630 |  |  | KX761617 | KX761714 |  |  |
|  | D17 | depa644 |  |  | KX761619 | KX761716 | 30.8233S; 50.5637W | BHCB 87286 |
|  |  | depa645 |  | x | KX761618 | KX761715 |  |  |
|  |  | depa646 |  |  | KX761618 | KX761715 |  |  |
|  |  | depa649 |  |  | KX761618 | KX761715 |  |  |
|  |  | depa650 |  |  | KX761618 | KX761715 |  |  |
|  |  | depa651 |  |  | KX761618 | KX761715 |  |  |
|  |  | depa652 |  |  | KX761618 | KX761715 |  |  |
|  |  | depa653 |  |  | KX761618 | KX761715 |  |  |
|  |  | depa654 |  |  | KX761618 | KX761715 |  |  |
|  |  | depa655 |  |  | KX761618 | KX761715 |  |  |
|  |  | depa667 |  |  | KX761618 | KX761715 |  |  |
|  |  | depa669 |  |  | KX761618 | KX761715 |  |  |
|  | D18 | depa741 |  |  | KX761618 | KX761715 | 30.9375S; 50.7393W | BHCB 87286 |
|  |  | depa747 |  |  | KX761618 | KX761715 |  |  |
|  |  | depa750 |  |  | KX761618 | KX761715 |  |  |
|  |  | depa751 |  |  | KX761617 | KX761714 |  |  |
|  |  | depa752 |  |  | KX761618 | KX761715 |  |  |
|  |  | depa753 |  |  | KX761618 | KX761715 |  |  |
|  |  | depa754 |  |  | KX761618 | KX761715 |  |  |
|  |  | depa755 |  |  | KX761618 | KX761715 |  |  |
|  |  | depa756 |  |  | KX761618 | KX761715 |  |  |
|  |  | depa763 |  |  | KX761618 | KX761715 |  |  |
|  |  | depa774 |  |  | KX761618 | KX761715 |  |  |
|  | D19 | depa856 |  |  | KX761618 | KX761715 | 31.1091S; 50.9011W | BHCB104894 |
|  |  | depa858 |  |  | KX761618 | KX761715 |  |  |
|  |  | depa860 |  |  | KX761618 | KX761715 |  |  |
|  |  | depa861 |  |  | KX761618 | KX761715 |  |  |
|  |  | depa862 |  |  | KX761618 | KX761715 |  |  |
|  |  | depa880 |  |  | KX761618 | KX761715 |  |  |
|  |  | depa882 |  |  | KX761618 | KX761715 |  |  |
|  |  | depa896 |  |  | KX761618 | KX761715 |  |  |
|  | D20 | depa961 |  |  | KX761626 | KX761723 | 31.2377S; 51.0095W | BHCB104896 |
|  |  | depa964 |  |  | KX761618 | KX761715 |  |  |
|  |  | depa966 |  |  | KX761618 | KF035653 |  |  |
|  |  | depa971 |  |  | KX761618 | KX761715 |  |  |
|  |  | depa972 |  |  | KX761618 | KX761715 |  |  |
|  |  | depa973 |  |  | KX761618 | KX761715 |  |  |
|  |  | depa974 |  |  | KX761626 | KX761723 |  |  |
|  |  | depa975 |  | x | KX761618 | KX761715 |  |  |
|  |  | depa976 |  |  | KX761626 | KX761723 |  |  |
|  |  | depa977 |  |  | KX761618 | KX761715 |  |  |
|  |  | depa980 |  |  | KX761618 | KX761715 |  |  |
|  | D21 | depa983 |  |  | KX761618 | KX761715 | 31.6667S; 51.4258W | BHCB 104894 |
|  |  | depa984 |  |  | KX761626 | KX761723 |  |  |
|  |  | depa986 |  |  | KX761618 | KX761715 |  |  |
|  |  | depa987 |  |  | KX761618 | KX761715 |  |  |
|  |  | depa988 |  |  | KX761618 | KX761715 |  |  |
|  |  | depa991 |  |  | KX761618 | KX761715 |  |  |
|  |  | depa992 |  |  | KX761618 | KX761715 |  |  |
|  |  | depa993 |  |  | KX761618 | KX761715 |  |  |
|  |  | depa996 |  |  | KX761618 | KX761715 |  |  |
|  | D22 | depa1000 |  |  | KX761627 | KF035655 | 32.1253; 52.1741W | BHCB 104904 |
|  |  | depa1001 |  |  | KX761627 | KX761724 |  |  |
|  |  | depa1006 |  |  | KX761627 | KX761724 |  |  |
|  |  | depa1007 |  | x | NA | NA |  |  |
|  |  | depa1009 |  |  | KX761618 | KX761715 |  |  |
|  |  | depa1012 |  |  | KX761628 | KX761725 |  |  |
|  |  | depa1013 |  |  | KX761628 | KX761725 |  |  |
|  |  | depa1015 |  |  | KX761627 | KX761724 |  |  |
|  |  | depa1016 |  |  | KX761627 | KX761724 |  |  |
|  |  | depa1017 |  |  | KX761627 | KX761724 |  |  |
|  | D23 | depa1033 |  |  | KX761629 | KX761726 | 32.5168S; 52.4993W | BHCB 104901 |
|  |  | depa1037 |  |  | KX761630 | KX761727 |  |  |
|  |  | depa1041 |  |  | KX761631 | KX761728 |  |  |
|  |  | depa1044 |  |  | KX761630 | KX761727 |  |  |
|  |  | depa1049 |  |  | KX761630 | KX761727 |  |  |
|  | D24 | depa1059 |  |  | KX761618 | KX761715 | 32.6047S; 52.4917W | BHCB 104843 |
|  |  | depa1061 |  |  | KX761632 | KX761729 |  |  |
|  |  | depa1062 |  |  | KX761632 | KX761729 |  |  |
|  |  | depa1065 |  |  | KX761618 | KX761715 |  |  |
|  | D25 | depa1151 | x |  | KX761618 | KX761715 | 33.6371S; 53.2225W | BHCB 104846 |
|  |  | depa1152 |  |  | KX761618 | KX761715 |  |  |
|  |  | depa1153 | x |  | NA | NA |  |  |
|  |  | depa1154 | x |  | NA | NA |  |  |
|  |  | depa1156 |  |  | KX761618 | KX761715 |  |  |
|  |  | depa1160 | x |  | KX761618 | KX761715 |  |  |
|  |  | depa1162 | x |  | KX761618 | KX761715 |  |  |
|  |  | depa1164 | x |  | KX761618 | KX761715 |  |  |
|  |  | depa1168 |  |  | KX761618 | KX761715 |  |  |
|  | D26 | depa1194 |  | x | KX761619 | KX761716 | 33.6723S; 53.2697W | BHCB 104851 |
|  | D27 | depa1256 |  |  | KX761618 | KX761715 | 31.8668S; 52.2714W | BHCB 104851 |
|  |  | depa1259 |  |  | KX761627 | KX761724 |  |  |
|  |  | depa1260 |  |  | KX761618 | KX761715 |  |  |
|  |  | depa1261 |  |  | KX761627 | KX761724 |  |  |
|  |  | depa1265 |  |  | KX761618 | KX761715 |  |  |
|  |  | depa1266 |  |  | KX761618 | KX761715 |  |  |
|  |  | depa1269 |  |  | KX761627 | KX761724 |  |  |
|  | D28 | depa1296 |  |  | KX761633 | KX761730 | 31.3767S; 51.9531W | BHCB 104861 |
|  |  | depa1300 |  |  | KX761633 | KX761730 |  |  |
|  |  | depa1301 |  |  | KX761633 | KX761730 |  |  |
|  |  | depa1302 |  |  | KX761618 | KX761715 |  |  |
|  |  | depa1303 |  |  | KX761618 | KX761715 |  |  |
|  |  | depa1305 |  |  | KX761618 | KX761715 |  |  |
|  |  | depa1306 |  |  | KX761618 | KX761715 |  |  |
|  |  | depa1308 |  |  | KX761618 | KX761715 |  |  |
|  |  | depa1313 |  |  | KX761618 | KX761715 |  |  |
|  |  | depa1314 |  | x | KX761618 | KX761715 |  |  |
|  |  | depa1317 |  |  | KX761618 | KX761715 |  |  |
|  |  | depa1320 |  |  | KX761618 | KX761715 |  |  |
|  | D29 | depa1326 |  |  | KX761620 | KX761717 | 29.6253S; 49.9356W | BHCB 104861 |
|  |  | depa1327 |  |  | KX761620 | KX761717 |  |  |
|  |  | depa1328 |  |  | KX761623 | KX761720 |  |  |
|  |  | depa1329 |  |  | KX761623 | KX761720 |  |  |
|  |  | depa1330 |  |  | KX761620 | KX761717 |  |  |
|  |  | depa1332 |  |  | KX761623 | KX761720 |  |  |
|  | D30 | depa1370 |  | x | KX761623 | KX761720 | 29.6439; 49.9479W | BHCB 104861 |
|  |  | depa1371 | x |  | KX761623 | KX761720 |  |  |
|  |  | depa1372 | x |  | KX761620 | KX761717 |  |  |
|  |  | depa1376 | x |  | NA | NA |  |  |
|  |  | depa1377 | x |  | KX761620 | KX761717 |  |  |
|  |  | depa1379 | x |  | KX761620 | KX761717 |  |  |
|  | D31 | depa1395 |  |  | KX761617 | KX761714 | 28.0214S; 48.62155W | BHCB 104857 |
|  |  | depa1397 |  |  | KX761617 | KX761714 |  |  |
|  |  | depa1399 |  |  | KX761617 | KX761714 |  |  |
|  |  | depa1400 |  |  | KX761617 | KX761714 |  |  |
|  |  | depa1401 |  |  | KX761617 | KX761714 |  |  |
|  |  | depa1403 |  |  | KX761617 | KX761714 |  |  |
|  |  | depa1404 |  |  | KX761617 | KX761714 |  |  |
|  |  | depa1405 |  |  | KX761617 | KX761714 |  |  |
|  |  | depa1413 |  |  | KX761617 | KX761714 |  |  |
|  |  | depa1417 |  |  | KX761617 | KX761714 |  |  |
|  | D33 | depa1447 |  |  | KX761634 | KX761731 |  | BHCB 104857 |
|  |  | depa1448 |  |  | KX761634 | KX761731 |  |  |
|  |  | depa1449 |  |  | KX761635 | KX761732 |  |  |
|  |  | depa1450 |  |  | KX761634 | KX761731 |  |  |
|  |  | depa1451 |  |  | KX761634 | KX761731 |  |  |
|  |  | depa1452 |  |  | KX761634 | KX761731 |  |  |
|  |  | depa1453 |  |  | KX761635 | KX761732 |  |  |
|  | D34 | depa1575 |  |  | KX761617 | KX761714 | 28.4604S; 48.7655W | BHCB 104857 |
|  | D35 | depa1576 |  |  | KX761617 | KX761714 | 28.8238S; 49.2176W | BHCB 104857 |
|  |  | depa1577 |  |  | KX761617 | KX761714 |  |  |
|  |  | depa1578 |  |  | KX761617 | KX761714 |  |  |
|  |  | depa1579 |  |  | KX761617 | KX761714 |  |  |
|  |  | depa1580 |  |  | KX761636 | KX761733 |  |  |
|  |  | depa1581 |  |  | KX761617 | KX761714 |  |  |
|  |  | depa1582 |  |  | KX761617 | KX761714 |  |  |
|  |  | depa1583 |  |  | KX761617 | KX761714 |  |  |
|  |  | depa1584 |  |  | KX761617 | KX761714 |  |  |
|  |  | depa1585 |  |  | KX761617 | KX761714 |  |  |
|  |  | depa1586 |  |  | KX761617 | KX761714 |  |  |
|  |  | depa1587 |  |  | KX761617 | KX761714 |  |  |
|  |  | depa1588 |  |  | KX761617 | KX761714 |  |  |
|  |  | depa1589 |  |  | KX761617 | KX761714 |  |  |
|  |  | depa1590 |  |  | KX761617 | KX761714 |  |  |
|  | D36 | depa1591 |  |  | KX761617 | KX761714 | 28.8420S; 49.4305W | BHCB 104857 |
|  |  | depa1592 |  |  | KX761617 | KX761714 |  |  |
|  |  | depa1594 |  |  | KX761617 | KX761714 |  |  |
|  |  | depa1596 |  |  | KX761617 | KX761714 |  |  |
|  |  | depa1597 |  |  | KX761617 | KX761714 |  |  |
|  |  | depa1598 |  |  | KX761637 | KX761734 |  |  |
|  |  | depa1600 |  |  | KX761638 | KX761735 |  |  |
|  |  | depa1601 |  |  | KX761617 | KX761714 |  |  |
|  |  | depa1602 |  |  | KX761617 | KX761714 |  |  |
|  |  | depa1603 |  |  | KX761638 | KX761735 |  |  |
|  |  | depa1604 |  |  | KX761617 | KX761714 |  |  |
|  | D37 | depa1605 |  |  | KX761617 | KX761714 | 28.9925S; 49.4166W | BHCB 104857 |
|  |  | depa1606 |  |  | KX761617 | KX761714 |  |  |
|  |  | depa1607 |  |  | KX761617 | KX761714 |  |  |
|  |  | depa1608 |  |  | KX761617 | KX761714 |  |  |
|  |  | depa1609 |  |  | KX761617 | KX761714 |  |  |
|  |  | depa1610 |  |  | KX761617 | KX761714 |  |  |
|  |  | depa1611 |  |  | KX761617 | KX761714 |  |  |
|  |  | depa1613 |  |  | KX761617 | KX761714 |  |  |
|  | D38 | depa1614 |  |  | KX761618 | KX761715 | 33.9111S; 53.5124W | BHCB 104857 |
|  |  | depa1615 |  |  | KX761618 | KX761715 |  |  |
|  |  | depa1616 |  |  | KX761618 | KX761715 |  |  |
|  |  | depa1617 |  |  | KX761618 | KX761715 |  |  |
|  |  | depa1619 |  |  | KX761639 | KX761736 |  |  |
|  |  | depa1620 |  |  | KX761618 | KX761715 |  |  |
|  |  | depa1621 |  |  | KX761618 | KX761715 |  |  |
|  |  | depa1622 |  |  | KX761618 | KX761715 |  |  |
|  |  | depa1623 |  |  | KX761618 | KX761715 |  |  |
|  | D39 | depa1641 |  |  | KX761617 | KX761714 | 28.4612S; 48.7681W | BHCB 104857 |
|  | D40 | depa1649 |  |  | KX761636 | KX761733 | 28.4734S; 48.7676W | BHCB 104857 |
|  |  | depa1650 |  |  | KX761617 | KX761714 |  |  |
|  |  | depa1651 |  |  | KX761617 | KX761714 |  |  |
|  |  | depa1654 |  |  | KX761617 | KX761714 |  |  |
|  |  | depa1655 |  |  | KX761636 | KX761733 |  |  |
|  |  | depa1656 |  |  | KX761617 | KX761714 |  |  |
|  |  | depa1657 |  |  | KX761617 | KX761714 |  |  |
|  |  | depa1658 |  |  | KX761617 | KX761714 |  |  |
|  |  | depa1659 |  |  | KX761617 | KX761714 |  |  |
|  | D41 | depa1673 |  |  | KX761623 | KX761720 |  | BHCB 79845 |
|  |  | depa1676 |  |  | KX761640 | KX761737 |  |  |
|  |  | depa1678 |  |  | KX761623 | KX761720 |  |  |
|  |  | depa1679 |  |  | KX761623 | KX761720 |  |  |
|  | D42 | depa1680 |  |  | KX761641 | KX761738 |  | BHCB 79845 |
|  |  | depa1681 |  |  | KX761642 | KX761739 |  |  |
|  |  | depa1682 |  |  | KX761620 | KX761717 |  |  |
|  | D43 | depa1685 |  |  | KX761640 | KX761737 |  | BHCB 79845 |
|  | D44 | depa1687 |  |  | KX761616 | KX761713 | 28.2019S; 48.6908W | BHCB 79845 |
|  |  | depa1688 |  |  | KX761643 | KX761740 |  |  |
|  | D45 | depa1691 |  |  | KX761617 | KX761714 | 29.1850S; 49.6136W | BHCB 79846 |
|  |  | depa1692 |  |  | KX761617 | KX761714 |  |  |
|  |  | depa1693 |  |  | KX761617 | KX761714 |  |  |
|  |  | depa1694 |  |  | KX761617 | KX761714 |  |  |
|  |  | depa1695 |  |  | KX761617 | KX761714 |  |  |
|  |  | depa1699 |  |  | KX761617 | KX761714 |  |  |
|  | D46 | depa1702 |  |  | KX761617 | KX761714 |  | BHCB 79845 |
|  |  | depa1705 |  |  | KX761617 | KX761714 |  |  |
|  | D47 | depa1697 |  |  | KX761616 | KX761713 |  | BHCB 79845 |
|  | D48 | depa1706 |  |  | KX761636 | KX761733 |  | BHCB 79845 |
|  |  | depa1707 |  |  | KX761636 | KX761733 |  |  |
|  | D49 | depa1709 |  |  | KX761616 | KX761713 | 29.3814S; 49.7658W | BHCB 79852 |
|  | D50 | depa1710 |  |  | KX761617 | KX761714 |  | BHCB 79852 |
|  |  | deoa1711 |  |  | KX761617 | KX761714 |  |  |
|  | D51 | depa1712 |  |  | KX761644 | KX761741 |  | BHCB 79852 |
|  |  | depa1714 |  |  | KX761645 | KX761742 |  |  |
|  |  | depa1718 |  |  | KX761644 | KX761741 |  |  |
|  |  | depa1720 |  |  | KX761623 | KX761720 |  |  |
|  | D52 | depa1728 |  |  | KX761623 | KX761720 | 29.4319S; 49.7969W | BHCB 79852 |
|  |  | depa1729 |  |  | KX761640 | KX761737 |  |  |
|  |  | depa1730 |  |  | KX761623 | KX761720 |  |  |
|  | D53 | depa1731 |  |  | KX761623 | KX761720 |  | BHCB 79852 |
|  |  | depa1732 |  |  | KX761623 | KX761720 |  |  |
|  |  | depa1733 |  |  | KX761623 | KX761720 |  |  |
|  | D54 | depa1739 |  |  | KX761649 | KX761746 | 29.6125S; 49.9344W | BHCB 79852 |
|  | D55 | depa1737 |  |  | KX761640 | KX761737 | 29.6125S; 49.9344W | BHCB 79852 |
|  | D56 | depa1742 |  |  | KX761618 | KX761715 |  | BHCB 79852 |
|  | D57 | depa1744 |  |  | KX761633 | KX761730 | 31.7664S; 52.2536W | BHCB 87264 |
|  | D58 | depa1747 |  |  | KX761633 | KX761730 | 30.5433S; 50.4128W | BHCB 87264 |
|  | D59 | depa1751 |  |  | KX761646 | KX761743 | 30.7025S; 50.5614W | BHCB 87264 |
|  | D60 | depa1752 |  |  | KX761618 | KX761715 |  | BHCB 87264 |
|  | D61 | depa1753 |  |  | KX761647 | KX761744 | 30.8433S; 50.6817W | BHCB 87264 |
|  | D62 | depa1754 |  |  | KX761618 | KX761715 |  | BHCB 87264 |
|  |  | depa1755 |  |  | KX761618 | KX761715 |  |  |
|  | D63 | depa1756 |  |  | KX761633 | KX761730 | 31.0103S; 50.8194W | BHCB 87264 |
|  |  | depa1757 |  |  | KX761650 | KX761747 |  |  |
|  | D64 | depa1758 |  |  | KX761640 | KX761737 |  | BHCB 87264 |
|  | D65 | depa1759 |  |  | KX761620 | KX761717 |  | BHCB 87264 |
|  | D66 | depa1760 |  |  | KX761623 | KX761720 |  | BHCB 87264 |
|  | D67 | depa1761 |  |  | KX761620 | KX761717 |  | BHCB 87264 |
|  | D68 | depa1762 |  |  | KX761617 | KX761714 |  | BHCB 87264 |
|  | D69 | depa1763 |  |  | KX761648 | KX761745 |  | BHCB 87264 |
|  | D70 | inte205 |  |  | KX761640 | KX761737 |  | BHCB 87264 |
| *Petunia inflata* R. E. Fr. | I1 | inf1 | x |  | KX761651 | KX761748 | 27.2500; 53.8667W | BHCB 87295 |
|  |  | inf2 |  |  | KX761651 | KX761748 |  |  |
|  | I2 | inf3 |  |  | KX761652 | KX761749 | 28.2996S; 54.2635W | BHCB 87295 |
|  |  | inf4 |  | x | KX761652 | KX761749 |  |  |
|  |  | inf5 |  |  | KX761653 | KX761750 |  |  |
|  |  | inf6 |  |  | KX761652 | KX761749 |  |  |
|  |  | inf7 |  |  | KX761652 | KX761749 |  |  |
|  | I3 | inf8 | x |  | NA | NA | 28.4567S; 55.1262W | BHCB 114603 |
|  |  | inf9 | x |  | KX761654 | KX761751 |  |  |
|  |  | inf10 | x |  | KX761655 | KX761752 |  |  |
|  |  | inf11 | x |  | KX761655 | KX761752 |  |  |
|  |  | inf12 |  |  | KX761655 | KX761752 |  |  |
|  |  | inf13 | x |  | KX761654 | KX761751 |  |  |
|  |  | inf22 | x |  | NA | NA |  |  |
|  | I4 | inf27 |  |  | KX761653 | KX761750 | 28.2513S; 54.8113W | BHCB 114605 |
|  |  | inf28 |  |  | KX761653 | KX761750 |  |  |
|  |  | inf29 |  |  | KX761653 | KX761750 |  |  |
|  |  | inf30 |  |  | KX761653 | KX761750 |  |  |
|  |  | inf31 |  |  | KX761653 | KX761750 |  |  |
|  |  | inf32 |  | x | KX761653 | KX761750 |  |  |
|  | I5 | inf45 |  |  | KX761656 | KX761753 | 27.8820S; 55.0552W | BHCB 114608 |
|  |  | inf46 |  |  | KX761656 | KX761753 |  |  |
|  |  | inf47 |  | x | KX761656 | KX761753 |  |  |
|  |  | inf50 |  |  | KX761653 | KX761750 |  |  |
|  | I6 | inf61 | x |  | KX761657 | KX761754 | 27.8379S; 54.6344W | BHCB 114610 |
|  |  | inf62 | x |  | KX761658 | KX761755 |  |  |
|  |  | inf63 |  |  | KX761658 | KX761755 |  |  |
|  |  | inf64 | x |  | NA | NA |  |  |
|  |  | inf65 | x |  | KX761658 | KX761755 |  |  |
|  |  | inf66 | x |  | KX761657 | KX761754 |  |  |
|  |  | inf68 | x |  | NA | NA |  |  |
|  |  | inf70 | x |  | NA | NA |  |  |
|  |  | inf71 |  | x | KX761659 | KX761756 |  |  |
|  |  | inf73 | x |  | NA | NA |  |  |
|  |  | inf74 | x |  | NA | NA |  |  |
|  |  | inf75 | x |  | NA | NA |  |  |
|  | I7 | inf88 |  | x | KX761660 | KX761757 | 28.1669S; 55.7211W | BHCB156818 |
|  | I8 | inf92 |  | x | KX761661 | KX761758 | 28.0000S; 56.1000W | BHCB156818 |
|  | I9 | inf98 |  | x | KX761662 | KX761759 | 27.4061S; 53.9128W | BHCB156818 |
|  |  | inf99 |  | x | KX761663 | KX761760 |  |  |
|  |  | inf100 |  | x | KX761664 | KX761761 |  |  |
|  | I10 | teri63 |  |  | KX761653 | KX761750 | 28.3832S; 54.0402W | BHCB002714 |
|  |  | teri64 |  |  | KX761653 | KX761750 |  |  |
|  |  | teri65 |  | x | KX761653 | KX761750 |  |  |
|  |  | teri66 |  |  | KX761653 | KX761750 |  |  |
|  |  | teri67 |  |  | KX761653 | KX761750 |  |  |
|  |  | teri68 |  |  | KX761668 | KX761765 |  |  |
| *Petunia interior* T. Ando and Hashim. | T1 | teri2 |  |  | KX761665 | KX761762 | 28.9311S; 52.3791W | BHCB 114596 |
|  |  | teri3 |  |  | KX761665 | KX761762 |  |  |
|  |  | teri4 |  |  | KX761665 | KX761762 |  |  |
|  |  | teri5 |  |  | KX761665 | KX761762 |  |  |
|  |  | teri6 |  |  | KX761665 | KX761762 |  |  |
|  | T2 | teri9 |  |  | KX761666 | KX761763 | 28.8798S; 52.4295W | BHCB 114596 |
|  |  | teri10 |  |  | KX761666 | KX761763 |  |  |
|  |  | teri11 |  |  | KX761666 | KX761763 |  |  |
|  |  | teri12 |  |  | KX761665 | KX761762 |  |  |
|  |  | teri14 |  |  | KX761665 | KX761762 |  |  |
|  | T3 | teri21 |  |  | KX761667 | KX761764 | 28.3373S; 53.5672W | BHCB 114598 |
|  |  | teri22 |  |  | KX761667 | KX761764 |  |  |
|  |  | teri23 |  |  | KX761667 | KX761764 |  |  |
|  |  | teri24 |  |  | KX761667 | KX761764 |  |  |
|  |  | teri25 |  |  | KX761667 | KX761764 |  |  |
|  |  | teri26 |  |  | KX761653 | KX761750 |  |  |
|  |  | teri27 |  |  | NA | NA |  |  |
|  |  | teri28 | x |  | NA | NA |  |  |
|  |  | teri30 | x |  | NA | NA |  |  |
|  |  | teri31 | x |  | NA | NA |  |  |
|  |  | teri32 | x |  | NA | NA |  |  |
|  |  | teri33 | x |  | NA | NA |  |  |
|  |  | teri34 | x |  | NA | NA |  |  |
|  |  | teri35 | x |  | NA | NA |  |  |
|  | T4 | teri43 |  |  | KX761653 | KX761750 | 28.3181S; 53.6177W | BHCB 114599 |
|  |  | teri44 |  |  | KX761653 | KX761750 |  |  |
|  |  | teri45 |  |  | KX761653 | KX761750 |  |  |
|  |  | teri48 |  |  | KX761653 | KX761750 |  |  |
|  | T5 | teri85 |  |  | KX761653 | KX761750 | 28.3473S; 54.2750W | BHCB 114601 |
|  |  | teri86 |  |  | KX761653 | KX761750 |  |  |
|  |  | teri87 |  |  | KX761669 | KX761766 |  |  |
|  |  | teri88 |  |  | KX761669 | KX761766 |  |  |
|  |  | teri89 |  |  | KX761669 | KX761766 |  |  |
|  |  | teri90 |  |  | KX761653 | KX761750 |  |  |
|  | T6 | teri110 |  | x | KX761653 | KX761750 | 28.4063S; 54.6910W | BHCB114602 |
|  |  | teri111 |  |  | KX761653 | KX761750 |  |  |
|  |  | teri112 | x |  | KX761653 | KX761750 |  |  |
|  |  | teri113 |  |  | KX761653 | KX761750 |  |  |
|  |  | teri114 | x |  | KX761653 | KX761750 |  |  |
|  |  | teri115 |  |  | KX761653 | KX761750 |  |  |
|  |  | teri117 | x |  | NA | NA |  |  |
|  |  | teri118 | x |  | NA | NA |  |  |
|  |  | teri120 | x |  | NA | NA |  |  |
|  |  | teri122 | x |  | NA | NA |  |  |
|  |  | teri123 | x |  | NA | NA |  |  |
|  |  | teri124 | x |  | NA | NA |  |  |
|  | T7 | teri130 | x |  | KX761667 | KX761764 | 27.7671S; 53.8162W | BHCB 114611 |
|  |  | teri131 |  |  | KX761667 | KX761764 |  |  |
|  |  | teri132 |  |  | KX761670 | KX761767 |  |  |
|  |  | teri133 |  |  | KX761667 | KX761764 |  |  |
|  |  | teri134 |  |  | KX761667 | KX761764 |  |  |
|  |  | teri135 |  |  | KX761667 | KX761764 |  |  |
|  | T8 | teri150 | x |  | KX761671 | KX761768 | 27.6277S; 53.5649W | BHCB 114612 |
|  |  | teri151 | x |  | KX761671 | KX761768 |  |  |
|  |  | teri152 | x |  | KX761671 | KX761768 |  |  |
|  |  | teri153 | x |  | KX761672 | KX761769 |  |  |
|  |  | teri154 | x |  | KX761671 | KX761768 |  |  |
|  |  | teri155 | x |  | KX761671 | KX761768 |  |  |
|  |  | teri56 | x |  | NA | NA |  |  |
|  |  | teri57 | x |  | NA | NA |  |  |
|  |  | teri158 | x | x | KX761673 | KX761770 |  |  |
|  |  | teri159 | x |  | NA | NA |  |  |
|  |  | teri160 | x |  | NA | NA |  |  |
|  |  | teri161 | x |  | NA | NA |  |  |
|  |  | teri162 | x |  | NA | NA |  |  |
|  |  | teri164 | x |  | NA | NA |  |  |
|  | T9 | teri180 |  |  | KX761674 | KX761771 | 27.0911S; 52.7910W | BHCB 114615 |
|  |  | teri181 |  |  | KX761674 | KX761771 |  |  |
|  |  | teri182 |  |  | KX761674 | KX761771 |  |  |
|  |  | teri183 |  | x | KX761675 | KX761772 |  |  |
|  |  | teri184 |  |  | KX761674 | KX761771 |  |  |
|  |  | teri185 |  |  | KX761674 | KX761771 |  |  |
|  | T10 | teri202 |  |  | KX761674 | KX761771 | 26.9523S; 52.5119W | BHCB 114616 |
|  |  | teri203 |  | x | KX761676 | KX761773 |  |  |
|  |  | teri204 |  |  | KX761674 | KX761771 |  |  |
|  |  | teri205 |  |  | KX761674 | KX761771 |  |  |
|  |  | teri206 |  | x | KX761674 | KX761771 |  |  |
|  |  | teri207 |  |  | KX761674 | KX761771 |  |  |
|  | T11 | teri221 |  | x | KX761677 | KX761774 | 26.2650S; 53.6569W | JRS 5120 |
|  | T12 | teri222 |  | x | KF280774 | KF280830 | 26.6313S; 54.1046W | JRS 5123 |
|  | T13 | teri223 |  |  | KX761677 | KX761774 | 26.2994S; 58.7750W | BHCB 156817 |
|  | T14 | teri226 |  | x | KX761678 | KX761775 | 27.2584S; 53.9806W | BHCB 156817 |
|  |  | teri227 |  | x | KX761679 | KX761776 |  |  |
|  |  | teri228 |  | x | KX761660 | KX761757 |  |  |
|  |  | teri229 |  |  | KX761680 | KX761777 |  |  |
|  |  | teri236 |  |  | KX761681 | KX761778 |  |  |
|  |  | teri238 |  |  | KX761682 | KX761779 |  |  |
|  |  | teri239 |  |  | KX761681 | KX761778 |  |  |
|  |  | teri240 |  |  | KX761683 | KX761780 |  |  |
|  |  | teri241 |  |  | KX761681 | KX761778 |  |  |
|  |  | teri242 |  |  | KX761684 | KX761781 |  |  |
|  |  | teri243 |  |  | KX761681 | KX761778 |  |  |
|  |  | teri244 |  |  | KX761683 | KX761780 |  |  |
|  |  | teri245 |  |  | KX761683 | KX761780 |  |  |
|  |  | teri246 |  |  | KX761681 | KX761778 |  |  |
|  |  | teri247 |  |  | KX761681 | KX761778 |  |  |
|  |  | teri248 |  |  | KX761681 | KX761778 |  |  |
|  |  | teri249 |  |  | KX761683 | KX761780 |  |  |
|  | T15 | teri251 |  | x | KX761679 | KX761776 | 27.2336S; 53.9783W | BHCB 156816 |
|  |  | teri253 |  |  | KX761679 | KX761776 |  |  |
|  |  | teri254 |  |  | KX761679 | KX761776 |  |  |
|  |  | teri255 |  |  | KX761679 | KX761776 |  |  |
|  |  | teri258 |  |  | KX761679 | KX761776 |  |  |
|  |  | teri259 |  |  | KX761679 | KX761776 |  |  |
|  |  | teri262 |  |  | KX761679 | KX761776 |  |  |
| *P. integrifolia* ssp. *integrifolia* (Hook.) Schinz and Thell. | G1 | inte01 |  |  | KX761634 | KX761731 | 30.2847S; 53.1328W | BHCB 79854 |
|  |  | inte02 |  | x | NA | NA | 30.2847S; 53.1328W |  |
|  |  | inte04 |  |  | KX761685 | KX761782 |  |  |
|  |  | inte06 |  |  | KX761634 | KX761731 |  |  |
|  | G2 | inte07 |  | x | KX761685 | KX761782 | 30.3686S; 53.3631W | BHCB 79854 |
|  | G3 | inte08 |  |  | KX761686 | KX761783 | 30.4833S; 53.3678W | BHCB 79854 |
|  |  | inte09 |  |  | KX761686 | KX761783 |  |  |
|  | G4 | inte10 |  |  | KX761687 | KX761784 | 30.5536S; 53.5203W | BHCB 79854 |
|  | G5 | inte12 |  | x | KX761688 | KX761785 | 30.6372S; 53.5511W | BHCB 79854 |
|  |  | inte13 |  |  | KX761688 | KX761785 |  |  |
|  |  | inte14 |  |  | KX761688 | KX761785 |  |  |
|  | G6 | inte15 | x |  | NA | NA | 30.3200S; 52.9261W | BHCB 79854 |
|  |  | inte16 | x |  | NA | NA |  |  |
|  |  | inte17 |  |  | KX761685 | KX761782 |  |  |
|  |  | inte20 | x | x | NA | NA |  |  |
|  | G7 | inte21 | x |  | KX761689 | KX761786 |  | BHCB 79854 |
|  |  | inte23 |  |  | KX761690 | KX761787 |  |  |
|  | G8 | inte24 |  | x | KX761691 | KX761788 | 30.3692S; 52.4297W | BHCB 79854 |
|  |  | inte25 | x |  | NA | NA |  |  |
|  |  | inte26 | x | x | KX761692 | KX761789 |  |  |
|  |  | inte27 | x | x | KX761693 | KX761790 |  |  |
|  |  | inte28 | x | x | KX761692 | KX761789 |  |  |
|  | G9 | inte50 |  |  | KX761634 | KX761731 | 30.1139S; 51.325W | BHCB 79854 |
|  | G10 | inte63 |  |  | KX761694 | KX761791 | 30.9183S; 54.7901W | BHCB 79863 |
|  | G11 | inte64 |  | x | KX761694 | KX761791 | 30.8409S; 55.0379W | BHCB 79865 |
|  | G12 | inte70 |  |  | KX761695 | KX761792 | 30.7925S; 55.2108W | BHCB 79866 |
|  |  | inte72 |  |  | KX761634 | KX761731 |  |  |
|  | G13 | inte73 |  |  | KX761696 | KX761793 | 30.8064S; 55.6173W | BHCB 79869 |
|  |  | inte74 |  | x | KX761696 | KX761793 |  |  |
|  | G14 | inte75 |  |  | KX761696 | KX761793 | 30.7908S; 55.7010W | BHCB79870 |
|  |  | inte78 |  |  | KX761696 | KX761793 |  |  |
|  | G15 | inte80 |  |  | KX761634 | KX761731 | 30.4841S; 56.2210W | BHCB 102117 |
|  |  | inte81 |  |  | KX761634 | KX761731 |  |  |
|  |  | inte82 |  |  | KX761634 | KX761731 |  |  |
|  |  | inte83 |  |  | KX761634 | KX761731 |  |  |
|  | G16 | inte86 |  |  | KX761634 | KX761731 | 30.3576S; 56.4565W | BHCB 79876 |
|  | G17 | inte90 |  | x | KX761697 | KX761794 | 29.8434S; 55.6625W | BHCB 79884 |
|  |  | inte91 |  | x | NA | NA |  |  |
|  |  | inte93 |  |  | KX761697 | KX761794 |  |  |
|  | G18 | inte94 |  |  | KX761697 | KX761794 | 29.8410S; 55.6675W | BHCB 79884 |
|  |  | inte95 |  |  | KX761697 | KX761794 |  |  |
|  |  | inte96 |  |  | KX761697 | KX761794 |  |  |
|  |  | inte97 |  |  | KX761697 | KX761794 |  |  |
|  | G19 | inte98 |  |  | KX761634 | KX761731 | 30.37250S; 53.6939W | BHCB 79885 |
|  |  | inte99 |  |  | KX761685 | KX761782 |  |  |
|  | G20 | inte101 |  |  | KX761634 | KX761731 | 30.7925S; 55.2108W | BHCB 79866 |
|  |  | inte102 |  |  | KX761634 | KX761731 |  |  |
|  | G21 | inte133 |  |  | KX761634 | KX761731 | 30.3039S; 51.4172W | BHCB 85204 |
|  | G22 | inte136 |  |  | KX761634 | KX761731 | 30.6397S; 51.5567W | BHCB 85205 |
|  | G23 | inte139 | x | x | KX761697 | KX761794 | 29.7902S; 55.7949W | BHCB 85218 |
|  | G24 | inte140 | x |  | KX761685 | KX761782 | 30.0725S; 52.3664W | BHCB 81676 |
|  |  | inte141 | x | x | NA | NA |  |  |
|  | G25 | inte142 |  | x | KX761686 | KX761783 | 30.3722S; 53.4289W | BHCB 85208 |
|  |  | inte143 |  |  | KX761686 | KX761783 |  |  |
|  |  | inte144 |  |  | KX761634 | KX761731 |  |  |
|  | G26 | inte299 |  |  | KX761634 | KX761731 | 30.1378S; 51.3178W | BHCB 104835 |
|  |  | inte300 |  |  | KX761634 | KX761731 |  |  |
|  |  | inte301 |  |  | KX761699 | KX761796 |  |  |
|  |  | inte303 |  |  | KX761700 | KX761797 |  |  |
|  | G27 | inte229 |  |  | KX761634 | KX761731 | 29.6756S; 53.9654W | BHCB 102085 |
|  |  | inte230 |  | x | NA | NA |  |  |
|  |  | inte231 |  |  | KX761634 | KX761731 |  |  |
|  |  | inte233 |  |  | KX761634 | KX761731 |  |  |
|  |  | inte235 |  |  | KX761634 | KX761731 |  |  |
|  | G28 | inte244 |  |  | KX761697 | KX761794 | 29.6349S; 54.2655W | BHCB 102098 |
|  |  | inte245 |  |  | KX761697 | KX761794 |  |  |
|  |  | inte246 | x |  | KX761697 | KX761794 |  |  |
|  | G29 | inte251 |  |  | KX761697 | KX761794 | 29.6385S; 54.3046W | BHCB 102098 |
|  |  | inte252 |  |  | KX761697 | KX761794 |  |  |
|  |  | inte253 |  |  | KX761697 | KX761794 |  |  |
|  |  | inte254 |  |  | KX761697 | KX761794 |  |  |
|  |  | inte255 |  |  | KX761697 | KX761794 |  |  |
|  |  | inte256 |  |  | KX761697 | KX761794 |  |  |
|  |  | inte257 |  |  | KX761697 | KX761794 |  |  |
|  | G30 | inte259 |  | x | KX761634 | KX761731 | 30.4372S; 56.3352W | BHCB 102115 |
|  |  | inte260 | x |  | KX761634 | KX761731 |  |  |
|  |  | inte261 | x |  | KX761634 | KX761731 |  |  |
|  |  | inte262 | x |  | KX761634 | KX761731 |  |  |
|  |  | inte263 | x |  | KX761634 | KX761731 |  |  |
|  |  | inte264 | x | x | NA | NA |  |  |
|  |  | inte265 | x |  | KX761634 | KX761731 |  |  |
|  |  | inte266 | x |  | NA | NA |  |  |
|  |  | inte268 | x |  | NA | NA |  |  |
|  |  | inte272 | x |  | NA | NA |  |  |
|  | G31 | inte276 |  |  | KX761701 | KX761798 | 30.5020S; 56.1967W | ICN155643 |
|  |  | inte278 |  |  | KX761634 | KX761731 |  |  |
|  |  | inte280 |  |  | KX761634 | KX761731 |  |  |
|  |  | inte281 |  |  | KX761634 | KX761731 |  |  |
|  |  | inte282 |  |  | KX761634 | KX761731 |  |  |
|  | G32 | inte289 |  |  | KX761695 | KX761792 | 30.8445S; 55.4460W | ICN155643 |
|  |  | inte291 |  |  | KX761696 | KX761793 |  |  |
|  |  | inte293 |  |  | KX761694 | KX761791 |  |  |
|  |  | inte294 |  |  | KX761694 | KX761791 |  |  |
|  |  | inte295 |  |  | KX761695 | KX761792 |  |  |
|  | G33 | inte321 |  |  | KX761702 | KX761799 | 29.5515S; 53.7907W | BHCB 104872 |
|  |  | inte322 |  |  | KX761702 | KX761799 |  |  |
|  |  | inte325 |  |  | KX761702 | KX761799 |  |  |
|  |  | inte326 |  |  | KX761702 | KX761799 |  |  |
|  | G34 | inte734 |  | x | NA | NA | 32.6939S; 58.1953W | Greppi1047 |
|  | G35 | inte964 |  |  | KX761703 | KX761800 | 30.5655S; 54.4386W | ICN155643 |
|  |  | inte965 |  |  | KX761704 | KX761801 |  |  |
|  |  | inte966 |  |  | KX761705 | KX761802 |  |  |
|  |  | inte967 |  |  | KX761706 | KX761803 |  |  |
|  |  | inte968 |  |  | KX761707 | KX761804 |  |  |
|  |  | inte969 |  |  | KX761707 | KX761804 |  |  |
|  |  | inte970 |  |  | KX761706 | KX761803 |  |  |
|  |  | inte971 |  |  | KX761707 | KX761804 |  |  |
|  |  | inte973 |  |  | KX761707 | KX761804 |  |  |
|  |  | inte974 |  |  | KX761707 | KX761804 |  |  |
|  | G36 | inte1071 |  |  | KX761708 | KX761805 | 31.3658S; 54.1084W | BHCB 151103 |
|  |  | inte1072 |  |  | KX761708 | KX761805 |  |  |
|  |  | inte1073 |  |  | KX761708 | KX761805 |  |  |
|  |  | inte1074 |  |  | KX761708 | KX761805 |  |  |
|  |  | inte1075 |  |  | KX761708 | KX761805 |  |  |
|  |  | inte1076 |  |  | KX761708 | KX761805 |  |  |
|  |  | inte1077 |  |  | KX761708 | KX761805 |  |  |
|  |  | inte1078 |  |  | KX761708 | KX761805 |  |  |
|  |  | inte1079 |  |  | KX761708 | KX761805 |  |  |
|  |  | inte1080 |  |  | KX761708 | KX761805 |  |  |
|  |  | inte1081 |  |  | KX761708 | KX761805 |  |  |
|  | G37 | inte1052 |  |  | KX761709 | KX761806 | 30.6630S; 51.3904W | BHCB 151103 |
|  |  | inte1054 |  |  | KX761709 | KX761806 |  |  |
|  | G38 | inte1065 |  |  | KX761634 | KX761731 | 30.9053S; 51.4918W | BHCB 151103 |
|  | G39 | depa1421 |  |  | KX761634 | KX761731 |  | BHCB 151103 |
|  |  | depa1422 |  |  | KX761634 | KX761731 |  |  |
|  |  | depa1423 |  |  | KX761634 | KX761731 |  |  |
|  |  | depa1424 |  |  | KX761634 | KX761731 |  |  |
| *Calibrachoa excellens* |  |  |  |  | KM982204  KM982205  KM982206  KM982207  KM982208 | KM982288  KM982287  KM982286  KM982285  KM982284 |  | ICN 181348 |

*Genbank number for plastid sequences, number are per haplotype without codification. NA – not analyzed; x – individuals included in AFLP and/or SSR analyses; BHCB – herbarium from Federal University of Minas Gerais; ICN – herbarium from Federal University of Rio Grande do Sul; JRS – collector number João Renato Stehmann (Brazil); Greppi – collector number Julián A. Greppi (Argentina).
