# Supplementary material for: Multiple markers, niche modelling, and bioregions analyses to evaluate the genetic diversity of a plant species complex
Source: BMC Evol Biol. 2017 Nov 29;17:234. doi: 10.1186/s12862-017-1084-y (PMC5707870; doi:10.1186/s12862-017-1084-y)
Supplement: Supplementary file 5 — Collection sites used in the Environmental Niche Modelling (ENM) analysis to the taxa of Petunia integrifolia complex. (DOCX 47 kb) [file 12862_2017_1084_MOESM5_ESM.docx]

**Additional file 5: Table S2 -** Collection sites used in the Environmental Niche Modelling (ENM) analysis.

| taxa | Code | Coordinate | Locality | Voucher |
| --- | --- | --- | --- | --- |
| *Petunia bajeensis* | baje1 | -31.384469, -54.123275 | Brazil/RS/Bagé | BHCB 102127 |
|  | baje2 | -31.433330, -54.133330 | Brazil/RS | MOBOT_BR 2475431 |
|  | baje3 | -31.377222, -54.116944 | Brazil/RS/Bagé | BM000941561 |
|  | baje4 | -31.371389, -54.066667 | Brazil/RS/Bagé | BM000941562 |
|  | baje5 | -31.408611, -54.133889 | Brazil/RS/Bagé | BM000941563 |
|  | baje6 | -31.299167, -54.047222 | Brazil/RS/Bagé | BM000941564 |
|  | baje7 | -31.360556, -54.108056 | Brazil/RS/Bagé | BM000941565 |
|  | baje8 | -31.373333, -54.113056 | Brazil/RS/Bagé | BM000941566 |
|  | baje9 | -31.440556, -54.138056 | Brazil/RS/Bagé | BM000583299 |
|  | baje10 | -31.331400, -54.106900 | Brazil/RS/Bagé | MBM0240689 |
|  | baje11 | -31.409756, -54.134936 | Brazil/RS/Bagé | BHCB002711 |
|  | baje12 | -30.982800, -54.673100 | Brazil/RS/Dom Pedrito | BHCB013185 |
|  | baje13 | -31.331400, -54.106900 | Brazil/RS/Bagé | ICN0148303 |
| *Petunia integrifolia* ssp*. integrifolia* | inte1 | -29.684200, -53.806900 | Brazil/RS/Santa Maria | UEC 156177 |
|  | inte2 | -30.235000, -51.575700 | Brazil/RS/São Jerônimo | NHM-LONDON-BOT BM000941595 |
|  | inte3 | -30.772222, -52.651389 | Brazil/RS/Encruzilhada do Sul | NHM-LONDON-BOT BM000941596 |
|  | inte4 | 30.526111, -52.266667 | Brazil/RS/Dom Feliciano | NHM-LONDON-BOT BM000941599 |
|  | inte5 | -31.574722, -53.309167 | Brazil/RS/Pinheiro Machado | NHM-LONDON-BOT BM000941600 |
|  | inte6 | 31.532222, -53.502222 | Brazil/RS/Pinheiro Machado | NHM-LONDON-BOT BM000941603 |
|  | inte7 | -30.039200, -52.893900 | Brazil/RS/Cachoeira do Sul | BHCB75132 |
|  | inte8 | -30.191400, -52.373600 | Brazil/RS/Pantano Grande | CESJ 45173 |
|  | inte9 | -30.160600, -53.565300 | Brazil/RS/São Sepé | BHCB17416 |
|  | inte10 | -30.512200, -53.491400 | Brazil/RS/Caçapava do Sul | BHCB156820 |
|  | inte11 | -29.760300, -51.147200 | Brazil/RS/São Leopoldo | HUSC6266 |
|  | inte12 | -30.258300, -54.914200 | Brazil/RS/Rosário do Sul | HUSC8751 |
|  | inte13 | -30.000300, -53.499200 | Brazil/RS/Formigueiro | UEC61420 |
|  | inte14 | -30.077200, -51.729200 | Brazil/RS/Arroio dos Ratos | BHCB38687 |
|  | inte15 | -30.813100, -53.895000 | Brazil/RS/Lavras do Sul | UEC77971 |
|  | inte16 | -30.126700, -52.047500 | Brazil/RS/Minas do Leão | BHCB38686 |
|  | inte17 | -30.387500, -56.451400 | Brazil/RS/Quaraí | BHCB51616 |
|  | inte18 | -31.331400, -54.106900 | Brazil/RS/Bajé | MBM259489 |
|  | inte19 | -31.578300, -53.381100 | Brazil/RS/Pinheiro Machado | MPUC14187 |
|  | inte20 | -29.550300, -55.131100 | Brazil/RS/ São Francisco de Assis | ICN153670 |
|  | inte21 | -29.466900, -51.961400 | Brazil/RS/Lajeado | HVAT4270 |
|  | inte22 | -30.113900, -51.325000 | Brazil/RS/Guaíba | BHCB 79854 |
|  | inte23 | -30.039444, -52.893889 | Brazil/RS/Cachoeira do Sul | MPUC1366 |
|  | inte24 | -30.512500, -53.491389 | Brazil/RS/Caçapava do Sul | MPUC14178 |
|  | inte25 | -30.872500, -53.136389 | Brazil/RS/Santana da Boa Vista | MPUC14181 |
|  | inte26 | -30.101167, -51.328056 | Brazil/RS/Guaíba | MPUC14182 |
|  | inte27 | -30.889444, -55.532778 | Brazil/RS/Sta. do Livramento | MPUC14184 |
|  | inte28 | -31.395000, -52.675600 | Brazil/RS/Canguçu | MPUC14188 |
|  | inte29 | -30.000600, -53.499200 | Brazil/RS/Formigueiro | UEC61420 |
|  | inte30 | -29.688600, -51.460800 | Brazil/RS/Montenegro | NYBG_BR656303 |
|  | inte31 | -30.833333, -52.875556 | Brazil/RS/Santana da Boa Vista | BHCB111957 |
|  | inte32 | -30.081100, -51.023300 | Brazil/RS/Viamão | BHCB127260 |
|  | inte33 | -29.883600, -54.825000 | Brazil/RS/Cacequi | BHCB117017 |
|  | inte34 | -31.853406, -58.320078 | Argentina/Entre Rios | BHCB127294 |
|  | inte35 | -30.907222, -57.691667 | Uruguay/Salto | BHCB136828 |
|  | inte36 | -30.777619, -55.700083 | Brazil/RS | BHCB140462 |
|  | inte37 | -30.677492, -55.858889 | Brazil/RS | BHCB140463 |
|  | inte38 | -30.460081, -56.278083 | Brazil/RS | BHCB140464 |
|  | inte39 | -29.859722, -55.946111 | Brazil/RS | BHCB140465 |
|  | inte40 | -29.906583, -55.556472 | Brazil/RS | BHCB140466 |
|  | inte41 | -29.982161, -55.422306 | Brazil/RS | BHCB140467 |
|  | inte42 | -30.180372, -55.105639 | Brazil/RS/Rosário do Sul | BHCB140468 |
|  | inte43 | -30.356511, -54.156111 | Brazil/RS/São Gabriel | BHCB140469 |
|  | inte44 | -30.375889, -53.710833 | Brazil/RS/São Sepé | BHCB140470 |
|  | inte45 | -31.516667, -55.683333 | Uruguay/Tacuarembó/Rocha | BHCB143125 |
|  | inte46 | -33.150000, -57.133333 | Uruguay/Flores | BHCB143126 |
|  | inte47 | -29.973833, -57.688333 | Argentina/Entre Rios | BHCB143945 |
|  | inte48 | -31.365750, -54.108417 | Brazil/RS/Bagé | BHCB151103 |
|  | inte49 | -30.360833, -53.634167 | Brazil/RS/São Sepé | BHCB156801 |
|  | inte50 | -30.890800, -55.532800 | Brazil/RS/Sta do Livramento | BHCB38681 |
|  | inte51 | -30.915000, -51.497800 | Brazil/RS/Arambaré | BHCB 99809 |
|  | inte52 | -30.586944, -56.031667 | Brazil/RS/Sta do Livramento | BHCB156805 |
|  | inte53 | -30.784722, -55.685278 | Brazil/RS/Sta do Livramento | BHCB156806 |
|  | inte54 | -30.753611, -56.023333 | Brazil/RS/Sta do Livramento | BHCB156809 |
|  | inte55 | -29.634722, -54.265278 | Brazil/RS/São Pedro do Sul | RB436939 |
|  | inte56 | -30.907222, -57.691667 | Argentina/Corrientes | RB496863 |
|  | inte57 | -30.871900, -53.115300 | Brazil/RS/Sta da Boa Vista | UFP23795 |
|  | inte58 | -30.218889, -51.404167 | Brazil/RS/Guaíba | HUEFS75273 |
|  | inte59 | -30.219167, -51.404167 | Brazil/RS/Guaíba | HUEFS75272 |
|  | inte60 | -31.891389, -58.240833 | Argentina/Entre Ríos/Colón | HUEFS211307 |
|  | inte61 | -30.543900, -52.521900 | Brazil/RS/Encruzilhada do Sul | UEC156174 |
|  | inte62 | -29.226900, -53.681700 | Brazil/RS/Júlio de Castilhos | UEC65791 |
|  | inte63 | -29.959200, -51.722200 | Brazil/RS/São Jerônimo | HAS17696 |
|  | inte64 | -30.336400, -54.320000 | Brazil/RS/São Gabriel | HAS20235 |
|  | inte65 | -30.091499, -51.687617 | Brazil/RS/Guaíba | HAS780 |
|  | inte66 | -30.855200, -55.495762 | Brazil/RS | HAS960 |
|  | inte67 | -29.547778, -54.252222 | Brazil/RS/São Pedro do Sul | ICN153079 |
|  | inte68 | -29.645833, -54.382500 | Brazil/RS/São Pedro do Sul | ICN155644 |
|  | inte69 | -29.634722, -54.265278 | Brazil/RS/São Pedro do Sul | ICN155647 |
|  | inte70 | -29.783100, -55.791900 | Brazil/RS/Alegrete | ICN155985 |
|  | inte71 | -31.684722, -56.050000 | Uruguay/Tacuarembó | ICN158370 |
|  | inte72 | -31.437778, -55.659722 | Uruguay/Rivera | ICN158372 |
|  | inte73 | -31.145278, -52.825000 | Brazil/RS/Canguçu | ICN158619 |
|  | inte74 | -30.859722, -51.849722 | Brazil/RS/Camaquã | ICN158622 |
|  | inte75 | -30.572222, -52.201389 | Brazil/RS/Dom Feliciano | ICN158624 |
|  | inte76 | -30.668611, -52.638056 | Brazil/RS/Encruzilhada do Sul | ICN158625 |
|  | inte77 | -30.761667, -53.923611 | Brazil/RS/Lavras do Sul | ICN158629 |
|  | inte78 | -30.805833, -52.569444 | Brazil/RS/Encruzilhada do Sul | ICN158634 |
|  | inte79 | -30.845833, -51.874722 | Brazil/RS/Camaquã | ICN158635 |
|  | inte80 | -30.695833, -54.081111 | Brazil/RS/Lavras do Sul | ICN158636 |
|  | inte81 | -31.558056, -53.403056 | Brazil/RS/Pinheiro Machado | ICN158637 |
|  | inte82 | -30.802778, -53.897222 | Brazil/RS/Lavras do Sul | ICN158638 |
|  | inte83 | -31.268889, -52.792500 | Brazil/RS/Pinheiro Machado | ICN158648 |
|  | inte84 | -30.284167, -53.131944 | Brazil/RS/Cachoeira do Sul | ICN181339 |
|  | inte85 | -29.688600, -51.461100 | Brazil/RS/Montenegro | PACA-AGP26635 |
|  | inte86 | -29.701700, -51.241900 | Brazil/RS/Portão | PACA-AGP43508 |
|  | inte87 | -29.824700, -51.148300 | Brazil/RS/Sapucaia do Sul | PACA-AGP57446 |
|  | inte88 | -30.284722, -53.132778 | Brazil/RS/Cachoeira do Sul |  |
|  | inte89 | -30.368611, -53.363056 | Brazil/RS/Caçapava do Sul |  |
|  | inte90 | -30.483333, -53.367778 | Brazil/RS/Caçapava do Sul |  |
|  | inte91 | -30.553611, -53.520278 | Brazil/RS/Caçapava do Sul |  |
|  | inte92 | -30.637222, -53.551111 | Brazil/RS/Caçapava do Sul |  |
|  | inte93 | -30.320000, -52.926111 | Brazil/RS/Cachoeira do Sul |  |
|  | inte94 | -30.369167, -52.429722 | Brazil/RS |  |
|  | inte95 | -30.918334, -54.790056 | Brazil/RS/Dom Pedrito | BHCB79863 |
|  | inte96 | -30.840935, -55.037907 | Brazil/RS/Dom Pedrito | BHCB79865 |
|  | inte97 | -30.792489, -55.210756 | Brazil/RS/Sta do Livramento | BHCB79866 |
|  | inte98 | -30.303889, -51.417222 | Brazil/RS/Pelotas | BHCB85204 |
|  | inte99 | -30.639722, -51.556667 | Brazil/RS/Pelotas | BHCB85205 |
|  | inte100 | -29.790166, -55.794862 | Brazil/RS/Alegrete | BHCB85218 |
|  | inte101 | -30.072500, -52.366389 | Brazil/RS/Pantano Grande | BHCB81676 |
|  | inte102 | -30.372222, -53.428889 | Brazil/RS/Caçapava do Sul | BHCB85208 |
|  | inte103 | -30.137778, -51.317778 | Brazil/RS/Guaíba | BHCB104835 |
|  | inte104 | -29.675646, -53.965354 | Brazil/RS/São Pedro do Sul | BHCB102085 |
|  | inte105 | -29.634895, -54.265508 | Brazil/RS/São Pedro do Sul |  |
|  | inte106 | -29.638458, -54.304625 | Brazil/RS/São Pedro do Sul | BHCB102098 |
|  | inte107 | -30.437230, -56.335209 | Brazil/RS/Quaraí | BHCB102115 |
|  | inte108 | -30.501962, -56.196722 | Brazil/RS/Quaraí | ICN155643 |
|  | inte109 | -30.844502, -55.446008 | Brazil/RS/Sta do Livramento |  |
|  | inte110 | -29.551510, -53.790676 | Brazil/RS/Itaara | BHCB104872 |
|  | inte111 | -30.565521, -54.438593 | Brazil/RS/Dom Pedrito |  |
|  | inte112 | -31.365762, -54.108426 | Brazil/RS/Bagé | BHCB151103 |
|  | inte113 | -30.662962, -51.390400 | Brazil/RS/Tapes |  |
|  | inte114 | -30.905300, -51.491810 | Brazil/RS/Arambaré |  |
|  | inte115 | -30.539167, -52.514722 | Brazil/RS/Encruzilhada do Sul | ICN158631 |
|  | inte116 | -30.484093, -56.220974 | Brazil/RS/Sta do Livramento | BHCB102117 |
|  | inte117 | -30.357579, -56.456486 | Brazil/RS/Quaraí-Uruguaiana | BHCB79876 |
|  | inte118 | -29.843372, -55.662486 | Brazil/RS/Alegrete | BHCB79884 |
|  | inte119 | -29.841027, -55.667464 | Brazil/RS/Alegrete |  |
|  | inte120 | -30.372500, -53.693889 | Brazil/RS/Caçapava do Sul | BHCB79885 |
|  | inte121 | -30.792489, -55.210756 | Brazil/RS/Sta do Livramento | BHCB79866 |
|  | inte122 | -30.806361, -55.617256 | Brazil/RS/Sta do Livramento | BHCB79869 |
|  | inte123 | -30.790815, -55.700996 | Brazil/RS/Sta do Livramento | BHCB79870 |
| *Petunia integrifolia* ssp*. depauperata* | depa1 | -27.522867, -48.417070 | Brazil/SC/Florianópolis | BHCB80104 |
|  | depa2 | -30.358976, -50.293133 | Brazil/RS/Quintão |  |
|  | depa3 | -30.248900, -50.260000 | Brazil/RS/Pinhal |  |
|  | depa4 | -30.247028, -50.260000 | Brazil/RS/Pinhal |  |
|  | depa5 | -30.261741, -50.260000 | Brazil/RS/Pinhal | BHCB99791 |
|  | depa6 | -30.340731, -50.267914 | Brazil/RS/Quintão |  |
|  | depa7 | -30.174144, -50.201514 | Brazil/RS/Cidreira |  |
|  | depa8 | -30.153852, -50.210000 | Brazil/RS |  |
|  | depa9 | -30.001564, -50.140000 | Brazil/RS/Imbé | BHCB87286 |
|  | depa10 | -29.867526, -50.080000 | Brazil/RS |  |
|  | depa11 | -29.858859, -50.080000 | Brazil/RS/Osório |  |
|  | depa12 | -29.431929, -49.797014 | Brazil/RS/Torres |  |
|  | depa13 | -29.813307, -50.242861 | Brazil/RS/Osório | BHCB 104842 |
|  | depa14 | -29.878135, -50.513161 | Brazil/RS/Osório |  |
|  | depa15 | -29.905202, -50.426189 | Brazil/RS/Sto Antonio da Patrulha |  |
|  | depa16 | -30.540921, -50.419559 | Brazil/RS/Mostardas |  |
|  | depa17 | -30.823259, -50.600000 | Brazil/RS/Mostardas |  |
|  | depa18 | -30.937462, -50.739340 | Brazil/RS/Mostardas |  |
|  | depa19 | -31.109093, -50.901125 | Brazil/RS/Mostardas | BHCB104894 |
|  | depa20 | -31.237741, -51.009536 | Brazil/RS/Tavares | BHCB104896 |
|  | depa21 | -31.666735, -51.425765 | Brazil/RS/Mostardas | BHCB104894 |
|  | depa22 | -32.125315, -52.174064 | Brazil/RS/Rio Grande | BHCB104904 |
|  | depa23 | -32.516827, -52.499355 | Brazil/RS/Rio Grande | BHCB104901 |
|  | depa24 | -32.604741, -52.491738 | Brazil/RS/Rio Grande | BHCB104843 |
|  | depa25 | -33.637060, -53.222544 | Brazil/RS/Santa Vitória do Palmar | BHCB104846 |
|  | depa26 | -33.672298, -53.269711 | Brazil/RS/Santa Vitória do Palmar |  |
|  | depa27 | -31.866841, -52.271374 | Brazil/RS/Rio Grande | BHCB104851 |
|  | depa28 | -31.376667, -51.953056 | Brazil/RS/São Lourenço do Sul | BHCB104861 |
|  | depa29 | -29.625278, -49.960000 | Brazil/RS/Capão Novo |  |
|  | depa30 | -29.642955, -49.947908 | Brazil/RS/Curumim |  |
|  | depa31 | -28.021391, -48.621537 | Brazil/SC/Garopaba | BHCB104857 |
|  | depa32 | -28.460410, -48.765495 | Brazil/SC/Laguna |  |
|  | depa33 | -28.823759, -49.217590 | Brazil/SC/Içara |  |
|  | depa34 | -28.842005, -49.430528 | Brazil/SC/Maracajá |  |
|  | depa35 | -28.992548, -49.416569 | Brazil/SC |  |
|  | depa36 | -33.911107, -53.512410 | Uruguay/La Coronilla |  |
|  | depa37 | -28.461164, -48.768120 | Brazil/SC/Laguna |  |
|  | depa38 | -28.473390, -48.767662 | Brazil/SC |  |
|  | depa39 | -28.201944, -48.690833 | Brazil/SC/Imbituba | BHCB79845 |
|  | depa40 | -29.185000, -49.613611 | Brazil/SC/Sombrio | BHCB79846 |
|  | depa41 | -29.381389, -49.765833 | Brazil/RS/Torres | BHCB79852 |
|  | depa42 | -29.431944, -49.796944 | Brazil/RS/Torres |  |
|  | depa43 | -29.612500, -49.934444 | Brazil/RS/Rolante |  |
|  | depa44 | -30.543333, -50.412778 | Brazil/RS/Mostardas |  |
|  | depa45 | -30.702500, -50.561389 | Brazil/RS/Mostardas |  |
|  | depa46 | -30.843333, -50.681667 | Brazil/RS/Mostardas |  |
|  | depa47 | -31.010278, -50.819444 | Brazil/RS/Mostardas |  |
|  | depa48 | -28.482500, -48.780800 | Brazil/SC/Laguna | MBM101958 |
|  | depa49 | -27.596700, -48.549200 | Brazil/SC/Florianópolis | MBM139957 |
|  | depa50 | -28.023300, -48.613300 | Brazil/SC/Garopaba | MBM139958 |
|  | depa51 | -29.335300, -49.726900 | Brazil/RS/Torres | MBM27344 |
|  | depa52 | -28.023300, -48.613300 | Brazil/SC/Garopaba | MBM139958 |
|  | depa53 | -31.707500, -52.164722 | Brazil/RS/Pelotas | ICN181859 |
| *Petunia* *inflata* | inf1 | -27.250000, -53.866667 | Brazil/RS/Derrubadas | BHCB87295 |
|  | inf2 | -28.299634, -54.263521 | Brazil/RS/Santo Ângelo |  |
|  | inf3 | -28.456669, -55.126199 | Brazil/RS/São Luiz Gonzaga | BHCB114603 |
|  | inf4 | -28.251318, -54.811347 | Brazil/RS/Rolador | BHCB114605 |
|  | inf5 | -27.881999, -55.055164 | Brazil/RS/Porto Xavier | BHCB114608 |
|  | inf6 | -27.837876, -54.634422 | Brazil/RS/Santo Cristo | BHCB114610 |
|  | inf7 | -28.166944, -55.721111 | Argetina/San Tomé/ |  |
|  | inf8 | -28.000000, -56.100000 | Argentina/ Corrientes/Virasoro |  |
|  | inf9 | -27.406111, -53.912778 | Brazil/RS/Derrubadas | BHCB156818 |
|  | inf10 | -27.400833, -55.601944 | Argentina/Misiones/San Ignacio | BHCB110831 |
|  | inf11 | -28.382778, -54.039722 | Brazil/RS/Coronel Barros | BHCB114600 |
|  | inf12 | -28.250833, -54.810833 | Brazil/RS/Rolador | BHCB114605 |
|  | inf13 | -28.026944, -55.636111 | Argentina/ Corrientes/Garruchos | BHCB127299 |
|  | inf14 | -27.303056, -55.531111 | Argentina/ Misiones/Candelaria | BHCB127302 |
|  | inf15 | -27.560000, -54.706944 | Argentina/ Misiones/Candelaria | BHCB127315 |
|  | inf16 | -28.083333, -55.650000 | Argentina/ Misiones/Azara | BHCB143098 |
|  | inf17 | -27.450000, -55.700000 | Argentina/ Misiones/ Candelaria | BHCB143100 |
|  | inf18 | -27.933333, -55.716667 | Argentina/ Misiones/Azara | BHCB143102 |
|  | inf19 | -28.066667, -55.983333 | Argentina/ Corrientes | BHCB143108 |
|  | inf20 | -28.400000, -51.483333 | Brazil/RS/Lagoa Vermelha | BHCB143132 |
|  | inf21 | -28.000000, -56.100000 | Argentina/ Corrientes/Virasoro | BHCB143944 |
|  | inf22 | -28.166833, -55.721333 | Argentina/ Misiones/Azara | BHCB143946 |
|  | inf23 | -27.466667, -55.483333 | Argentina/ Misiones/Candelaria | BHCB143951 |
|  | inf24 | -27.948333, -52.929444 | Brazil/RS/Sarandi | BHCB156812 |
|  | inf25 | -27.923889, -53.026111 | Brazil/RS/Barra Funda | BHCB156813 |
|  | inf26 | -27.779444, -53.436389 | Brazil/RS/Palmeira das Missões | BHCB156814 |
|  | inf27 | -28.299200, -54.263100 | Brazil/RS/Santo Ângelo | BHCB17519 |
|  | inf28 | -27.371100, -53.758300 | Brazil/RS/Tente Portela | ICN088924 |
|  | inf29 | -27.909200, -53.108100 | Brazil/RS/Novo Barreiro | ICN129146 |
|  | inf30 | -28.262800, -52.406700 | Brazil/RS/Passo Fundo | ICN140034 |
|  | inf31 | -28.168611, -51.667500 | Brazil/RS/Lagoa Vermelha | ICN141647 |
|  | inf32 | -28.023056, -52.232500 | Brazil/RS/Sertão | ICN141656 |
|  | inf33 | -27.644722, -53.284722 | Brazil/RS/Jaboticaba | ICN181358 |
|  | inf34 | -27.702222, -53.314444 | Brazil/RS/Boa Vista | ICN181359 |
| *Petunia interior* | teri1 | -28.931115, -52.379092 | Brazil/RS/Fontoura Xavier | BHCB114596 |
|  | teri2 | -28.879795, -52.429490 | Brazil/RS/Soledade |  |
|  | teri3 | -28.337349, -53.567184 | Brazil/RS/Panambí | BHCB114598 |
|  | teri4 | -28.318078, -53.617679 | Brazil/RS/Panambí | BHCB114599 |
|  | teri5 | -28.383162, -54.040163 | Brazil/RS/Coronel Barros | BHCB002714 |
|  | teri6 | -28.347275, -54.275022 | Brazil/RS/Entre Ijuís | BHCB 114601 |
|  | teri7 | -28.406253, -54.691043 | Brazil/RS/São Luiz Gonzaga | BHCB114602 |
|  | teri8 | -27.767102, -53.816160 | Brazil/RS/Santo Augusto | BHCB114611 |
|  | teri9 | -27.627731, -53.564865 | Brazil/RS/Dois Irmãos das Missões | BHCB114612 |
|  | teri10 | -27.091099, -52.790977 | Brazil/SC/Guatambú | BHCB114615 |
|  | teri11 | -26.952307, -52.511886 | Brazil/SC/Xaxim | BHCB114616 |
|  | teri12 | -26.265000, -53.656944 | Argentina/Misiones | BHCB127307 |
|  | teri13 | -26.631308, -54.104597 | Argentina/Misiones/San Pedro | BHCB127312 |
|  | teri14 | -27.258417, -53.980583 | Brazil/RS/Derrubadas | BHCB156817 |
|  | teri15 | -27.233583, -53.978306 | Brazil/RS/Derrubadas | BHCB156816 |
|  | teri16 | -27.223889, -52.668888 | Brazil/SC/Xapecó | NMNH-BOTANY_BR 3358971.2140453 |
|  | teri17 | -27.216660, -52.666660 | Brazil/SC/Xapecó | MOBOT_BR2475474 |
|  | teri18 | -27.029167, -52.634167 | Brazil/SC | NHM-LONDON-BOT BM000941577 |
|  | teri19 | -27.094444, -52.686667 | Brazil/SC | NHM-LONDON-BOT BM000941578 |
|  | teri20 | -27.738611, -53.836944 | Brazil/SC | NHM-LONDON-BOT BM000941579 |
|  | teri21 | -27.478100, -53.402500 | Brazil/RS/Seberi | SOLANACEAE_SOURCE_BR 37660 |
|  | teri22 | -27.363056, -52.786667 | Brazil/RS/Nonoai | SOLANACEAE_SOURCE_BR 37658 |
|  | teri23 | -26.436944, -53.230833 | Brazil/SC/Campo Erê | SOLANACEAE_SOURCE_BR 37659 |
|  | teri24 | -27.223889, -52.668889 | Brazil/SC/Chapecó | SOLANACEAE_SOURCE_BR 47520 |
|  | teri25 | -26.265000, -53.656944 | Argentina/Misiones | BHCB127307 |
|  | teri26 | -26.350000, -54.183333 | Argentina/Misiones/Eldorado | BHCB143950 |
|  | teri27 | -27.766944, -53.811944 | Brazil/RS/Esquina da Boa Vista | BHCB156815 |
|  | teri28 | -27.096400, -52.618300 | Brazil/SC/Chapecó | BHCB51619 |
|  | teri29 | -27.264700, -53.860800 | Brazil/RS/Derrubadas | BHCB87296 |
|  | teri30 | -26.872222, -52.399166 | Brazil/SC/Xanxerê | RB551100 |
|  | teri31 | -26.404167, -53.173889 | Brazil/SC/Campos Erê | R-TIPOS211336 |
|  | teri32 | -27.000000, -52.715000 | Brazil/SC/Chapecó | R-TIPOS211337 |
|  | teri33 | -26.410556, -53.187222 | Brazil/SC/Campos Erê | R-TIPOS211355 |
|  | teri34 | -26.396111, -53.135556 | Brazil/SC/Campos Erê | R-TIPOS211357 |
|  | teri35 | -26.576111, -52.333333 | Brazil/SC/Abelardo Luz | R-TIPOS211366 |
|  | teri36 | -26.365833, -53.271667 | Brazil/SC | R-TIPOS211390 |

Herbaria acronyms according to Thiers (2010) [117].
