# Supplementary material for: Multiple markers, niche modelling, and bioregions analyses to evaluate the genetic diversity of a plant species complex
Source: BMC Evol Biol. 2017 Nov 29;17:234. doi: 10.1186/s12862-017-1084-y (PMC5707870; doi:10.1186/s12862-017-1084-y)
Supplement: Supplementary file 6 — Plastid haplotypes observed for each species of the Petunia integrifolia complex. (DOCX 13 kb) [file 12862_2017_1084_MOESM6_ESM.docx]

**Additional file 6: Table S3 -** Plastid haplotypes observed for each species of the *Petunia integrifolia* complex.

| **Species** | **N** | **n** | **Haplotypes** |
| --- | --- | --- | --- |
| *P. bajeensis* | 3 | 28 | H1, H2, H3 |
| *P. integrifolia* ssp. i*ntegrifolia* | 39 | 126 | H8, H18, **H43**, H60, H61, H62, H63, H64, H65, H66, H67, H68, H69, H70, H71, H72, H73, H74, H75, H76, H77 |
| *P. integrifolia* ssp*. depauperata* | 70 | 320 | H4, H5, H6, H7, H9, H10, H11, H12, H13, H14, H15, H16, H17, H19, H20, H21, H22, H23, H24, H25, H26, H27, H28, H29, H30, H31 |
| *P. inflata* | 10 | 47 | H32, H33, **H34**, H35, H36, H37, H38, H39, H40, **H41**, H42, **H43**, H44 |
| *P. interior* | 15 | 105 | **H34**, **H41**, H45, H46, H47, H48, H49, H50, H51, H52, H53, H54, H55, H56, H57, H58, H59 |
| N – number of populations; n – total number of individuals; Haplotypes in bold correspond to sharing among species. | | | |
