# Supplementary material for: Multiple markers, niche modelling, and bioregions analyses to evaluate the genetic diversity of a plant species complex
Source: BMC Evol Biol. 2017 Nov 29;17:234. doi: 10.1186/s12862-017-1084-y (PMC5707870; doi:10.1186/s12862-017-1084-y)
Supplement: Supplementary file 8 — Diversity indices for the Petunia integrifolia complex based on microsatellite variation. (DOCX 17 kb) [file 12862_2017_1084_MOESM8_ESM.docx]

**Additional file8: Table S4 -** Diversity indices for the *Petunia integrifolia* complex based on microsatellite variation.

| Locus | *P. bajeensis* | | | | | *P. integrifolia* ssp. *integrifolia* | | | | | *P. integrifolia* ssp. *depauperata* | | | | | *P. inflata* | | | | | *P. interior* | | | | |
| --- | --- | --- | --- | --- | --- | --- | --- | --- | --- | --- | --- | --- | --- | --- | --- | --- | --- | --- | --- | --- | --- | --- | --- | --- | --- |
|  | A | AR | GD | *F_IS_* | EX | A | AR | GD | *F_IS_* | EX | A | AR | GD | *F_IS_* | EX | A | AR | GD | *F_IS_* | EX | A | AR | GD | *F_IS_* | EX |
| PID1D6 | 2 | 2.0 | 0.5 | -0.27 |  | 7 | 5.2 | 0.8 | 0.54 |  | 4 | 3.3 | 0.6 | 0.47 |  | 6 | 4.8 | 0.8 | 0.23 |  | 7 | 4.5 | 0.7 | 0.18 |  |
| PID1F1 | 3 | 2.9 | 0.6 | -0.24 |  | 3 | 2.5 | 0.5 | 0.54 |  | 3 | 2.7 | 0.5 | 0.25 |  | 5 | 3.9 | 0.7 | 0.76 |  | 10 | 5.0 | 0.8 | 0.63 | 5 |
| PID1G6 | 1 | 1.0 | 0.0 |  |  | 2 | 1.8 | 0.2 | 0.46 |  | 2 | 1.2 | 0.1 |  |  | 4 | 2.7 | 0.6 | 0.78 |  | 3 | 2.4 | 0.5 | 0.64 |  |
| PID3C4 | 4 | 3.3 | 0.6 | -0.10 |  | 5 | 3.5 | 0.7 | 0.14 |  | 8 | 4.6 | 0.8 | 0.14 | 2 | 6 | 3.4 | 0.6 | 0.45 |  | 7 | 4.2 | 0.7 | 0.44 | 1 |
| PID3G5 | 2 | 2.0 | 0.5 | -0.57 |  | 2 | 1.6 | 0.2 | -0.04 | 1 | 2 | 1.8 | 0.2 | -0.12 |  | 1 | 1.0 | 0.0 |  |  | 1 | 1.0 | 0.0 |  |  |
| PID3H7 | 2 | 1.9 | 0.3 | 1.00 |  | 2 | 1.7 | 0.2 | -0.07 |  | 2 | 2.0 | 0.4 | 0.22 |  | 3 | 2.6 | 0.6 | 1.00 |  | 3 | 2.7 | 0.6 | 0.93 | 1 |
| PID4C6 | 2 | 2.0 | 0.5 | -0.85 |  | 1 | 1.0 | 0.0 |  |  | 2 | 1.2 | 0.0 |  | 1 | 2 | 1.3 | 0.1 |  | 1 | 1 | 1.0 | 0.0 |  |  |
| **Average** | **2** | **2.2** | **0.4** |  |  | **3** | **2.4** | **0.4** |  |  | **3** | **2.4** | **0.4** |  |  | **4** | **2.8** | **0.5** |  |  | **5** | **3.0** | **0.5** |  |  |

A: allele number; AR: allele richness; GD: gene diversity; *F_IS_*: inbreeding coefficient; EX: exclusive alleles.
