# Supplementary material for: Multiple markers, niche modelling, and bioregions analyses to evaluate the genetic diversity of a plant species complex
Source: BMC Evol Biol. 2017 Nov 29;17:234. doi: 10.1186/s12862-017-1084-y (PMC5707870; doi:10.1186/s12862-017-1084-y)
Supplement: Supplementary file 9 — Microsatellite-based diversity indices for species of Petunia integrifolia complex. (DOCX 14 kb) [file 12862_2017_1084_MOESM9_ESM.docx]

**Additional file 9: Table S5 -** Microsatellite-based diversity indices for species of *Petunia integrifolia* complex.

| Locus | *P. bajeensis* | | | | *P. integrifolia* ssp *.integrifolia* | | | | *P. integrifolia* ssp. *depauperata* | | | | *P. inflata* | | | | *P. interior* | | | |
| --- | --- | --- | --- | --- | --- | --- | --- | --- | --- | --- | --- | --- | --- | --- | --- | --- | --- | --- | --- | --- |
|  | ***H_O_*** | ***H_E_*** | **PIC** | **NUL** | ***H_O_*** | ***H_E_*** | **PIC** | **NUL** | ***H_O_*** | ***H_E_*** | **PIC** | **NUL** | ***H_O_*** | ***H_E_*** | **PIC** | **NUL** | ***H_O_*** | ***H_E_*** | **PIC** | **NUL** |
| PID1D6 | 0.64 | 0.51 | 0.38 | -0.14 | 0.39 | 0.82 | 0.76 | 0.34 | 0.33 | 0.62 | 0.54 | 0.28 | 0.62 | 0.79 | 0.73 | 0.09 | 0.59 | 0.72 | 0.68 | 0.09 |
| PID1F1 | 0.80 | 0.63 | 0.53 | -0.12 | 0.23 | 0.51 | 0.42 | 0.35 | 0.39 | 0.52 | 0.45 | 0.1 | 0.18 | 0.73 | 0.65 | 0.60 | 0.28* | 0.76 | 0.72 | 0.48 |
| PID1G6 | 0.00 | 0.00 | 0.00 | - | 0.13 | 0.23 | 0.20 | 0.27 | 0.05 | 0.05 | 0.05 | -0.01 | 0.13 | 0.58 | 0.46 | 0.6 | 0.18 | 0.50 | 0.41 | 0.47 |
| PID3C4 | 0.67 | 0.70 | 0.53 | -0.05 | 0.59 | 0.68 | 0.61 | 0.03 | 0.65 | 0.75 | 0.70 | 0.08 | 0.32 | 0.57 | 0.51 | 0.29 | 0.40 | 0.72 | 0.67 | 0.29 |
| PID3G5 | 0.75 | 0.50 | 0.36 | -0.23 | 0.15 | 0.15 | 0.13 | -0.03 | 0.25 | 0.22 | 0.20 | -0.06 | 0.00 | 0.00 | 0.00 | - | 0.00 | 0.00 | 0.00 | - |
| PID3H7 | 0.00 | 0.29 | 0.24 | 0.98 | 0.19 | 0.18 | 0.16 | -0.04 | 0.30 | 0.39 | 0.31 | 0.11 | 0.00 | 0.54 | 0.44 | 0.2 | 0.04 | 0.56 | 0.47 | 0.87 |
| PID4C6 | 0.92 | 0.52 | 0.37 | -0.30 | 0.0 | 0.00 | 0.00 | - | 0.04 | 0.04 | 0.04 | -0.01 | 0.05 | 0.05 | 0.05 | - | 0.00 | 0.00 | 0.00 | - |

H_O_: observed heterozygosity; H_E_: expected heterozygosity; PIC: polymorphic information content; NUL: % of null alleles; * Hardy–Weinberg equilibrium deviation significance after Bonferroni’s correction (P = 0.05) at minimum expected frequency specified of 1.
