# Supplementary material for: Multiple markers, niche modelling, and bioregions analyses to evaluate the genetic diversity of a plant species complex
Source: BMC Evol Biol. 2017 Nov 29;17:234. doi: 10.1186/s12862-017-1084-y (PMC5707870; doi:10.1186/s12862-017-1084-y)
Supplement: Supplementary file 10 — AFLP-based genetic diversity of the Petunia integrifolia complex. (DOCX 12 kb) [file 12862_2017_1084_MOESM10_ESM.docx]

**Additional file 10: Table S6 -** AFLP-based genetic diversity of the *Petunia integrifolia* complex.

| **Species** | **N** | **# P_loc (%)** | **Hj** |
| --- | --- | --- | --- |
| *P. bajeensis* | 7 | 54.7 | 0.004 |
| *P. integrifolia* ssp. *integrifolia* | 16 | 57.3 | 0.005 |
| *P. integrifolia* ssp. *depauperata* | 8 | 52.4 | 0.005 |
| *P. inflata* | 9 | 60.0 | 0.005 |
| *P. interior* | 11 | 52.4 | 0.004 |

# P_loc (%) – percentage of polymorphic loci; Hj – gene diversity.
