# Supplementary material for: Multiple markers, niche modelling, and bioregions analyses to evaluate the genetic diversity of a plant species complex
Source: BMC Evol Biol. 2017 Nov 29;17:234. doi: 10.1186/s12862-017-1084-y (PMC5707870; doi:10.1186/s12862-017-1084-y)
Supplement: Supplementary file 11 — AUC values and standard deviations of the Ecological Niche Modeling. (DOCX 14 kb) [file 12862_2017_1084_MOESM11_ESM.docx]

**Additional file 11: Table S7 -** AUC values and standard deviations of the Ecological Niche Modelling.

| Niche modelling | *Petunia bajeensis* | *Petunia integrifolia* ssp. *integrifolia* | *Petunia integrifolia* ssp. *depauperata* | *Petunia inflata* | *Petunia interior* |
| --- | --- | --- | --- | --- | --- |
| Present | 0.994 (0.016) | 0.919 (0.028) | 0.986 (0.009) | 0.942 (0.023) | 0.973 (0.021) |
| Mid Holocene | 0.993 (0.017) | 0.921 (0.026) | 0.987 (0.009) | 0.939 (0.020) | 0.979 (0.011) |
| Last Glacial Maximum | 0.997 (0.008) | 0.930 (0.039) | 0.946 (0.037) | 0.963 (0.027) | 0.961 (0.031) |
| Last Interglacial | 0.997 (0.002) | 0.958 (0.010) | 0.987 (0.004) | 0.961 (0.011) | 0.979 (0.006) |
