# Supplementary material for: Multiple markers, niche modelling, and bioregions analyses to evaluate the genetic diversity of a plant species complex
Source: BMC Evol Biol. 2017 Nov 29;17:234. doi: 10.1186/s12862-017-1084-y (PMC5707870; doi:10.1186/s12862-017-1084-y)
Supplement: Supplementary file 12 — Selected bioregions for each species of the Petunia integrifolia complex. (DOCX 12 kb) [file 12862_2017_1084_MOESM12_ESM.docx]

**Additional file 12: Table S8.** Selected *Petunia integrifolia* complex bioregions.

| **Bioregion** | **Taxa** | **Occurrence points** | **Goodness-of-fit (GOF) Score** |
| --- | --- | --- | --- |
| 1 | *P. integrifolia* ssp. *integrifolia* | 215 | 1.00 |
|  | *P. integrifolia* ssp. *depauperata* | 41 | 0.96 |
|  | *P. inflata* | 2 | 0.07 |
| 2 | *P. interior* | 48 | 6.10 |
|  | *P. inflata* | 38 | 5.52 |
|  | *P. integrifolia* ssp. *integrifolia* | 30 | 0.63 |
| 3 | *P. bajeensis* | 37 | 7.92 |
|  | *P. integrifolia* ssp. *integrifolia* | 33 | 0.89 |
